# Compositionally and functionally distinct sinus microbiota in chronic rhinosinusitis patients have immunological and clinically divergent consequences

**DOI:** 10.1186/s40168-017-0266-6

**Published:** 2017-05-12

**Authors:** Emily K. Cope, Andrew N. Goldberg, Steven D. Pletcher, Susan V. Lynch

**Affiliations:** 1 Department of Otolaryngology, University of California, San Francisco, CA 94143 USA; 2 Division of Gastroenterology, Department of Medicine, University of California, San Francisco, CA 94143 USA; 3 Present Address: Pathogen and Microbiome Institute, Northern Arizona University, Flagstaff, AZ 86011 USA

**Keywords:** Microbiome, Sinus, Airway, Chronic rhinosinusitis, Cystic fibrosis, Colonization patterns, Predicted metagenome, Dirichlet state, Microbiota state, Microbiota

## Abstract

**Background:**

Chronic rhinosinusitis (CRS) is a heterogeneous disease characterized by persistent sinonasal inflammation and sinus microbiome dysbiosis. The basis of this heterogeneity is poorly understood. We sought to address the hypothesis that a limited number of compositionally distinct pathogenic bacterial microbiota exist in CRS patients and invoke discrete immune responses and clinical phenotypes in CRS patients.

**Results:**

Sinus brushings from patients with CRS (*n* = 59) and healthy individuals (*n* = 10) collected during endoscopic sinus surgery were analyzed using 16S rRNA gene sequencing, predicted metagenomics, and RNA profiling of the mucosal immune response. We show that CRS patients cluster into distinct sub-groups (DSI-III), each defined by specific pattern of bacterial co-colonization (permutational multivariate analysis of variance (PERMANOVA); *p* = 0.001, *r* ^2^ = 0.318). Each sub-group was typically dominated by a pathogenic family: *Streptococcaceae* (DSI), *Pseudomonadaceae* (DSII), *Corynebacteriaceae* [DSIII(a)], or *Staphylococcaceae* [DSIII(b)]*.* Each pathogenic microbiota was predicted to be functionally distinct (PERMANOVA; *p* = 0.005, *r* ^2^ = 0.217) and encode uniquely enriched gene pathways including ansamycin biosynthesis (DSI), tryptophan metabolism (DSII), two-component response [DSIII(b)], and the PPAR-γ signaling pathway [DSIII(a)]. Each is also associated with significantly distinct host immune responses; DSI, II, and III(b) invoked a variety of pro-inflammatory, T_H_1 responses, while DSIII(a), which exhibited significantly increased incidence of nasal polyps (Fisher’s exact; *p* = 0.034, relative risk = 2.16), primarily induced IL-5 expression (Kruskal Wallis; *q* = 0.045).

**Conclusions:**

A large proportion of CRS patient heterogeneity may be explained by the composition of their sinus bacterial microbiota and related host immune response—features which may inform strategies for tailored therapy in this patient population.

**Electronic supplementary material:**

The online version of this article (doi:10.1186/s40168-017-0266-6) contains supplementary material, which is available to authorized users.

## Background

The field of human microbiome research has profoundly altered our view of the diversity of human-associated microbes and encoded functions, and demonstrated that the microbiome co-varies with host health status [1–3]. In healthy subjects, a diverse bacterial microbiota colonizes the upper respiratory mucosal surface [4, 5], and the lower airways possess a low burden and diversity of bacteria [6]. In contrast, patients with chronic inflammatory airway disease exhibit opposing trends in bacterial diversity and burden and compositionally distinct mucosal microbiota, enriched for known or suspected pathogenic species, and related to features of pulmonary disease [1, 5, 7, 8]. Chronic rhinosinusitis (CRS), characterized by persistent inflammation of the sinonasal mucosa lasting at least 12 weeks, is a common and refractory respiratory disease [9, 10], not least because of the immunologic and clinical heterogeneity exhibited by these patients. Until recently, little was known of the microbiome of the sinus mucosa in either healthy subjects or diseased patients. However, several recent culture-independent studies have now demonstrated that loss of sinus microbiota diversity is a common feature of patients with CRS [5, 11, 12] and, independently, that greater pre-operative sinus microbiota diversity is associated with improved post operative outcomes [13]. Respiratory pathogens such as *Pseudomonas aeruginosa* or *Staphylococcus aureus* are commonly isolated from CRS patients [14], while pathobionts, such as *Corynebacterium tuberculostearicum*, also found to be enriched in CRS patients, have demonstrable capacity to induce sinus mucosal infection in murine models [5]. However, these pathogens do not exist in isolation, but in mixed-species mucosal microbiota, the composition and activities of which, we hypothesize, explain the substantial clinical and immunological heterogeneity observed in CRS patients.

Previous efforts to explain CRS patient heterogeneity have been based on clinical [15, 16], immunologic [17–20], or pathologic [21] endotypes, though these studies have been relatively small and focused on specific immune cell populations or clinical features. More recently airway studies have examined whether subject stratification based on microbiota composition offers an improved approach for understanding immunological or clinical phenotypic variation across populations. A large study (*n* = 234) of the infant nasopharyngeal microbiota identified six compositionally distinct microbiota, each dominated by a common respiratory bacterial genus and associated with significantly different relative risk for acute upper respiratory infection or development of asthma at 5 years [4]. Similarly, three compositionally and functionally distinct pathogenic lung microbiota have been described in HIV-infected pneumonia patients (*n* = 182), each co-associate with a specific host immune response profile and differ in mortality risk [22]. Moreover, predicted metabolic products characteristic of each of these three distinct pathogenic lower airway communities were found to be enriched in paired serum samples, indicating that the microbiome of the overtly colonized airway may actively contribute both to local and systemic immune and metabolic dysfunction. The capacity for meaningful stratification based on microbiota composition is perhaps most compelling in a recent study of 1-month-old infants (*n* = 130), who were divisible into three distinct gut microbiota states, one of which conferred a three-fold increased risk of atopy at the age of 2 years and asthma at the age of 4 years. The associated products of the high-risk microbiota induced CD4 + IL4+ cell population expansion and CD4 + CD25 + FoxP3+ suppression ex vivo [23]. Hence, several lines of investigation suggest that patient immunological status and clinical outcomes differ significantly based on the specific microbiota structure present. Given these observations, we hypothesized CRS patient heterogeneity may be explained by the presence of distinct pathogenic sinus microbiota that invoke discrete host immune responses and relate to clinical phenotypes. To address this hypothesis, we examined the sinus mucosal microbiome and parallel host immune responses of a cohort of CRS and healthy subjects and related these findings to clinical outcomes of nasal polyposis. We demonstrate the presence of distinct pathogenic sinus microbiota in CRS patients each predicted to encode unique functional attributes, which co-associate with specific innate and adaptive immune responses, and significantly different relative risk of nasal polyposis.

## Methods

### Study design

The UCSF Institutional Review Board (approval number 11-07750) approved this study. All participants were informed of the objectives of this study and signed a written consent form prior to their participation. Adult patients (*n* = 76) undergoing endoscopic sinus surgery (ESS) for CRS were enrolled at the University of California San Francisco. Samples from two patients did not yield PCR product, and five patients were removed from the analysis due to low sequence depth. Therefore, 69 subjects (10 healthy, 59 CRS patients) were included in downstream analysis (Table 1).

**Table 1:** Demographics of CRS patients and healthy subjects included in this study

Sample ID	Age	Gender	Disease	Pre-operative antibiotics <3 months	Lund-Mackay score	Polyp	Status
33	47	F	CRS+CF	Multiple (>3)	22	N	Included
36	27	F	CRS+CF	Multiple (>3)	18	Y	Included
39	28	M	CRS+CF	Multiple (>3)	13	N	Included
61	20	M	CRS+CF	Azithromycin	15	Y	Included
64	71	M	CRS+CF	Dicloxacillin	16	Y	Included
83	24	M	CRS+CF	Multiple	19	Y	Included
90	20	F	CRS+CF	Ceftrioxone	19	Y	Included
91	47	M	CRS+CF	Zithromax/dapsone	17	Y	Included
108	30	M	CRS+CF	Multiple (>3)	16	Y	Included
1	41	M	CRS	Augmentin	5	N	Included
7	39	F	CRS	Augmentin	12	N	Included
8	55	F	CRS+Asthma	Augmentin	23	Y	Included
15	55	M	CRS+Asthma	Clarithromycin	15	N	Included
16	51	M	CRS	Augmentin	22	N	Included
17	61	F	CRS+Asthma	Multiple (>3)	12	N	Included
22	58	M	CRS+Asthma	Bactrim	12	Y	Included
32	19	M	CRS	Augmentin	22	Y	Included
34	51	M	CRS+Asthma	Levofloxacin	5	N	Included
40	72	F	CRS	Tobramycin	9	N	Included
43	46	M	CRS	Augmentin	12	N	Included
44	62	M	CRS	Levofloxacin	20	Y	Included
54	71	M	CRS+Asthma	dicloxacillin	13	Y	<10,000 sequences/sample
55	68	F	CRS+Asthma	Clotrimazole	21	Y	Included
56	67	F	CRS	Augmentin/Bactrim	14	Y	Included
58	85	M	CRS	Ampicillin/sulbactam	ND^a^	ND	No PCR product
59	42	F	CRS+Asthma	Clindamycin	4	Y	Included
60	35	M	CRS	Multiple (>3)	16	N	Included
63	77	F	CRS	Levofloxacin	6	N	Included
80	58	M	CRS	Augmentin	8	N	Included
81	65	F	CRS+Asthma	Augmentin	10	N	Included
82	28	M	CRS	Augmentin	9	Y	Included
85	24	F	CRS+Asthma	Augmentin	4	N	Included
86	88	F	CRS	None	19	Y	Included
88	73	M	CRS	None	21	Y	Included
89	52	M	CRS	Clarithromycin	ND	ND	No PCR product
92	59	M	CRS	Multiple (>3)	13	N	Included
93	48	M	CRS	Azithromycin/augmentin	11	Y	Included
94	72	F	CRS+Asthma	Augmentin	8	N	Included
96	54	M	CRS	Cephalexin	11	Y	Included
97	52	M	CRS	Cetirizine	22	Y	Included
98	39	M	CRS+Asthma	Multiple (>3)	16	Y	Included
99	57	M	CRS	Levofloxacin	16	Y	Included
100	62	F	CRS	Bactrim	4	N	Included
101	27	M	CRS+Asthma	Azithromycin	ND	Y	Included
103	36	M	CRS	Augmentin	10	Y	Included
104	23	M	CRS	None	12	N	Included
105	18	M	CRS	None	21	N	Included
107	37	F	CRS	Cephalexin	7	Y	Included
109	71	M	CRS+Asthma	Augmentin	11	Y	Included
110	18	F	CRS	Multiple (>3)	16	N	Included
111	59	M	CRS	Augmentin	10	N	Included
112	48	F	CRS	Augmentin	9	N	Included
114	37	F	CRS	Clindamycin	1	N	Included
115	26	F	CRS	Augmentin	3	N	Included
117	33	M	CRS	Ciprofloxacin	19	N	Included
120	50	F	CRS+Asthma	Multiple (>3)	15	Y	Included
121	33	F	CRS	Augmentin	9	Y	Included
122	45	F	CRS+Asthma	Augmentin	21	Y	Included
123	74	M	CRS+Asthma	Augmentin	16	Y	Included
124	30	M	CRS+Asthma	Augmentin	6	N	<10,000 sequences/sample
126	43	F	CRS+Asthma	Augmentin	11	Y	<10,000 sequences/sample
128	69	M	CRS+Asthma	Bactrim	21	Y	Included
130	59	M	CRS+Asthma	Augmentin	17	Y	Included
132	48	M	CRS	Rifampin	13	N	<10,000 sequences/sample
143	61	M	CRS	Augmentin	ND	N	Included
30	38	M	Healthy	Topical bacitracin	ND	N	<10,000 sequences/sample
31	59	M	Healthy	Amoxicillin/azithromycin	ND	N	Included
131	59	F	Healthy^b^﻿	None	1	N	Included
CRS14	41	M	Healthy^b^	None	ND	N	Included
CRS15	39	M	Healthy	None	ND	N	Included
CRS16	37	F	Healthy	None	ND	N	Included
CRS17	46	F	Healthy	None	ND	N	Included
CRS18	46	M	Healthy	None	ND	N	Included
CRS19	31	F	Healthy	None	ND	N	Included
CRS20	18	F	Healthy	None	ND	N	Included
ctrl4	22	M	Healthy	None	ND	N	Included

^*a*^ *ND* not determined

^b^ Allergic rhinitis

### Patient enrollment and sample collection

Disease was clinically diagnosed according to the 2007 Rhinosinusitis Task Force guidelines [24], and severity was radiographically quantified using the Lund-Mackay Computed Tomography (CT) scoring system. All CRS patients had symptoms for more than 12 consecutive weeks and CT evidence of inflammation within a month of sampling for this study. Patient demographics are described in Table 1. Recent clinical history, sinonasal outcomes test (SNOT-20), and CT sinus review were collected and used to confirm CRS diagnosis. Recent antibiotic use and intraoperative antibiotic administrations were recorded at the time of sample collection. Co-morbidities, including physician-diagnosed asthma or cystic fibrosis (CF), were recorded. Sinus brushings were obtained for 11 control patients undergoing surgery for non-CRS etiologies including oral surgery, trans-sphenoidal pituitary surgery, or endoscopic cerebral spinal fluid leak repair. Endoscopically guided protected brushes (ConMed #149, NY) were used to collect mucosal samples of the diseased sinus by brushing each surface gently while rotating the brush five times. Each sample was immediately placed in 1 ml of RNA later, transferred to 4 °C for 24–48 h to permit the nucleic acid preservative to permeate cells prior to storage at −80 °C [25].

### DNA extraction

Nucleic acids were extracted as previously described using the AllPrep kit (Qiagen, CA), to purify DNA and RNA in parallel [5, 25]. Briefly, brushes were placed in Lysis Matrix B tubes in 600 μl Buffer RLT Plus with β-mercaptoethanol and bead beaten for 30 s at 5.5 m sec^−1^ for nucleic acid extraction per manufacturer’s protocol. DNA and RNA were quantified using a NanoDrop 2000 (ThermoFisher, CA). DNA concentrations were normalized to 50 ng μl^−1^ per sample for 16S rRNA gene sequence library preparation, described below.

### 16S rRNA gene library preparation

Barcoded primers 515 F/806R were used to amplify the V4 region of the 16S rRNA gene as previously described [26, 27]. Since double bands were present, one human mitochondrial band and a microbial 16S band, amplicons of the correct size (384 bp) were gel-extracted with a Qiagen Gel Extraction kit per manufacturer protocol. Purified PCR product was analyzed on Bioanalyzer (Aligent, CA, USA), quantified using the Qubit HS dsDNA kit (Invitrogen, CA, USA), and pooled at 25 ng per sample. The pooled library was quantified using the KAPA QPCR Illumina Library Quantification kit (KAPA Biosystems, MA, USA), diluted to 2 nM, denatured, and 5 pM was loaded onto the Illumina MiSeq cartridge (V2) in combination with a 15% (*v*/*v*) of denatured 12.5 pM PhiX spike-in. In addition to negative control extraction blanks, a mock community composed of equal genomic concentration (2 ng each per reaction) of *Escherichia coli* ATCC25922, *P. aeruginosa* ATCC27853, *C. tuberculostearicum* ATCC35692, *Lactobacillus sakei* ATCC15521, and *L. rhamnosus* ATCC53103 was also used to monitor runs.

### 16S rRNA gene sequence processing

Sequence analysis of 16S rRNA data was performed using Quantative Insights Into Microbial Ecology (QIIME) version 1.8.0 [28] and in the R environment. See Supplemental Methods for details.

### Sequence and statistical analyses

Since our rarefaction curves approached an asymptote (indicating adequate community coverage) at a sequence depth 10,055 sequences, and all but 5 samples were sequenced at least to this depth, the operational taxonomic unit (OTU) table was multiple rarefied to 10,055 high-quality, chimera-checked sequences per sample for subsequent analyses using a custom script (https://github.com/alifar76/MicroNorm). All subsequent analyses were performed on this rarefied table. UniFrac, Canberra, and Bray-Curtis dissimilarity matrices were generated in QIIME 1.8.0, and Principal Coordinates Analysis (PCoA) plots were used to visualize ordinations using emperor [29]. Permutational multivariate analysis of variance (PERMANOVA) using the adonis function in the R Vegan package was used to determine significance in dissimilarity matrices across samples by metadata categories (e.g., disease, Dirichlet state, antibiotic use, age, and disease severity [30, 31]). Faith’s phylogenetic diversity, number of unique OTUs (richness), and Pielou’s evenness were calculated and a permutational *t* test (999 Monte Carlo permutations) was used to determine changes in alpha diversity. When multiple comparisons were performed, we corrected for false discovery using the Benjamini-Hochberg method and reported the corrected *p* values as *q* values, a *q* < 0.05 was considered significant [32]. Changes in taxon relative abundance were determined per OTU using a zero-inflated negative binomial (https://github.com/alifar76/NegBinSig-Test) distribution on a regression model. Kruskal-Wallis was used to determine if statistically significant differences in OTU or Kyoto Encyclopedia of Genes and Genomes (KEGG) pathway abundances existed between more than two groups, such as healthy patients, CRS, and CRS+CF patients. To identify clusters, the Dirichlet-multinomial mixtures probabilistic community modeling was performed using the *DirichletMultinomial* package [33] in R with family-level taxonomy using absolute abundances of each family. The Laplace approximation was used to calculate model fit and to determine the number of components (clusters). Distinct sample clusters that represented the best model fit were termed Dirichlet states (DS). To determine whether DSIII could be separated into two phylogenetically distinct groups, hierarchical cluster analysis was performed on a weighted UniFrac distance matrix using an edited version of *pvclust* in R (code attached in Additional file 1, Additional file 2, Additional file 3, Additional file 4, Additional file 5). Kruskal-Wallis was used to determine whether host genes were significantly up- or down-regulated in disease. Statistical analysis was performed using R.

### Predicted metagenomics

Metagenome prediction from the closed-reference OTUs (greengenes 13_5) of the multiple rarefied OTU table was performed using the Phylogenetic Investigation of Communities by Reconstruction of Unobserved States (PICRUSt v. 1.0.0 [34]). QIIME 1.8.0 was used to analyze the predicted metagenomes. Differential abundances of pathways were tested using a Kruskal-Wallis test when comparing more than two groups or a three-model approach (negative binomial, zero-inflated negative binomial, or poisson distributions) applied on a regression to test pairwise comparisons. Model fit was determined using Akaike information criterion (AIC) values, and the associated statistic was reported (https://github.com/alifar76/NegBinSig-Test). Nearest Sequenced Taxon Index (NTSI) scores were calculated using the –a flag in metagenome_predictions.py. These represent the average branch length separating OTUs in a sample from a reference bacterial genome. A heatmap was constructed for KEGG categories that were enriched or depleted in each disease state using heatmap.3 in R. For visualization, read counts were normalized [log2(*x* + 1)] and scaled by row. See Additional file 6: Supplemental Methods for more detail.

### Quantitative PCR for bacterial burden and human gene expression

Quantitative PCR (qPCR) was used to quantify bacterial burden as a ratio to human beta-actin. See Supplemental Methods for primers and PCR conditions. A custom qPCR array was developed (SA Biosciences, Hilden, Germany) and used to quantify host gene expression using RNA extracted in parallel from patient sinus brushes. See Additional file 6: Supplemental Methods for gene targets and reverse transcriptase (RT) PCR conditions.

## Results

### Sinus mucosal microbiome perturbations characterize CRS and are related to disease status

Our cohort consisted of 76 subjects. Sixty-five were CRS patients and 11 were healthy subjects. CRS patients included those with concomitant lower airway disease i.e. cystic fibrosis (CRS+CF) or asthma (CRS+A; Table 1). Sinus brushing samples from 2 subjects yielded no 16S rRNA amplicons (both from CRS patients), and a further 5 samples were removed due to low sequence depth (<10,000 sequences/sample; *n* = 4 CRS and *n* = 1 healthy). Thus, 10 healthy individuals and 59 CRS patients were included in the analyses presented.

**Fig. 1:**
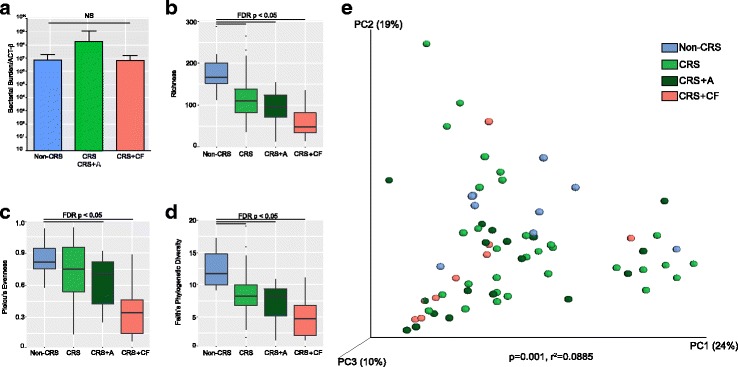
CRS patients (irrespective of lower airway disease status) exhibit similar total bacterial burden compared with healthy subjects; however, their microbiota exhibit significantly reduced richness, evenness and diversity. Comparative analyses of sinus microbiota **a** bacterial burden; **b** richness (permutation *t* test; CRS vs healthy *q* = 0.006; CRS+A vs healthy, *q* = 0.0015 CRS+CF vs healthy, *q* = 0.0015); **c** Pielou’s evenness (permutation *t* test; CRS vs healthy *q* = 0.132, CRS+A vs healthy, *q* = 0.015 and CRS+CF vs healthy, *q* = 0.003]), and **d** Faith’s phylogenetic diversity (permutation *t* test; CRS vs healthy, *q* = 0.007, CRS+A vs healthy, *q* = 0.003 and CRS+CF vs healthy, *q* = 0.003) indices using V4 16S rRNA amplicon sequencing of healthy, CRS, CRS+A and CRS+CF subjects. Values represent the median +/− 1.5 IQR. **e** Multivariate permutation testing of principal coordinate analysis using PERMANOVA based on a weighted UniFrac distance matrix of all samples indicates that disease status explains 8.9% of community composition variation (*p* = 0.001)

CRS patients exhibited signifcantly higher Lund McKay scores compared to healthy subjects﻿ (p<0.05), however amongst CRS patients,﻿ disease severity did not differ based on the presence or absence of concomitant lower airway disease (Tukey’s post hoc comparison; *p* > 0.05, Additional file 6: Figure S1A). Mucosal bacterial burden (based on total 16S rRNA copy number) was not significantly different across healthy and CRS patients (ANOVA; *p* = 0.781; Fig. 1a), consistent with previous reports [5, 12]. Also consistent with previous findings [5, 12] was the observation that compared with healthy subjects, CRS patients exhibited significantly lower microbiota richness, evenness, and diversity. Of note, diminished alpha diversity in CRS patients was more pronounced in those with concomitant lower airway disease (permutational *t* test; all *p* < 0.01; Fig. 1b–d). Multivariate analysis (PERMANOVA) of sinus bacterial beta diversity on a weighted UniFrac distance matrix was used to determine whether factors such as age, antimicrobial administration, polyposis, revision surgery (a complete list is provided in Table 2) explained the observed variation in community composition across all subjects (CRS and healthy), or exclusively within the CRS patients. Of these, only disease status (healthy, CRS, CRS+CF, CRS+A) was significantly related to beta diversity, but only explained a small portion of microbiota compositional variance and the effect size was small (PERMANOVA; *p* = 0.001, 8.9% of variation explained, Fig. 1e; Table 2).

**Table 2:** Multivariate analysis (PERMANOVA) of sinus bacterial beta diversity on a weighted UniFrac distance matrix

PERMANOVA (patient cohort)	r^2^	*p* value
***Dirichlet state (disease*** ^***a***^ ***)***	***0.326***	***0.001***
***Dirichlet state (all*** ^***b***^ ***)***	***0.318***	***0.001***
***Disease (all)***	***0.088***	***0.014***
Antibiotic use <3 months (disease)	0.036	0.052
Polyp presence/absence (disease)	0.025	0.152
Anatomic location (all)	0.039	0.185
Age bin 10 year (all)	0.118	0.196
Antibiotic class <3 months (all)	0.260	0.24
Age bin 5 year (all)	0.153	0.367
Antibiotic class <3 months (disease)	0.286	0.399
Anatomic location (disease)	0.0348	0.403
Age bin 10 year (disease)	0.119	0.473
Age bin 5 year (disease)	0.168	0.521
LMS Bin (low/medium/high) (disease)	0.046	0.554
Revision surgery (Y/N) (disease)	0.042	0.668
Age (disease)	0.674	0.799
Lund-Mackay score (LMS) (disease)	0.327	0.906
Age (all)	0.561	0.944

^a^CRS, CRS+CF, CRS+A

^b^Healthy, CRS, CRS+A, CRS+CF

Those in boldface are significant (the r2 value indicates the degree of community variance explained by the specific factor)

### Discrete pathogenic sinus microbiota exists in CRS patients

**Fig. 2:**
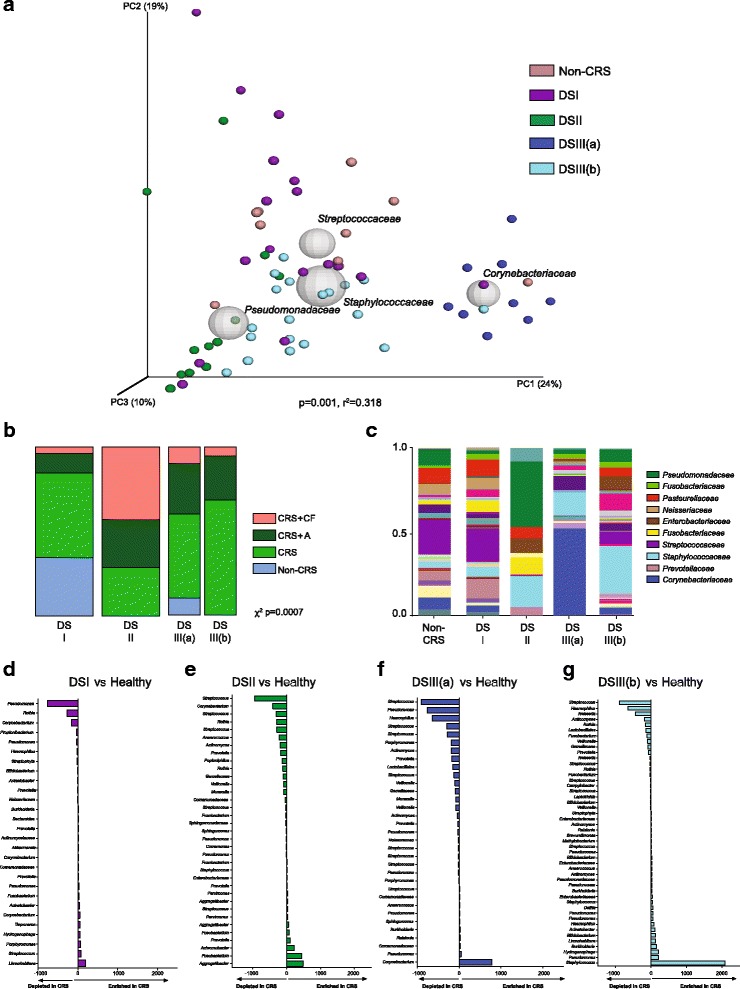
Dirichlet-multinomial mixtures modeling identifies microbial states that explain a large portion of variation in microbiota composition. **a** Multivariate permutation (PERMANOVA) testing of DS designation (I-III(b)) explains 31.8% of variation in sinus mucosal microbiota composition (*p* = 0.001). The most abundant family-level taxa are indicated. Size of sphere is proportional to the average relative abundance of each dominant taxon. **b** Distribution of co-morbidities (CF or physician-diagnosed asthma) signifcantly differ across microbiota states. DSI was represented by CRS patients and 9/10 of the healthy controls. DSII was enriched for CRS+CF and CRS+A patients, whereas DSIII was comprosed primarily of CRS patients without concomitant lower airway disease, and one healthy control subject (Chi-squared; *p* = 0.0007). **c** *Stacked bar chart* indicating the distribution of taxa in the sinuses grouped by DS. **d**–**g** Three-model testing of differential taxon abundance indicates that each DS is associated with enrichment for specific taxa and co-colonizers and depletion of microbiota associated with healthy individuals (ZINB; *p* < 0.05, *q* < 0.10)

We postulated that the microbiota dysbiosis exhibited by CRS patients does not represent a single state, but rather a gradient of dysbioses punctuated by a limited number of distinct pathogenic microbiota compositional states. We addressed this hypothesis through the application of an unbiased probabilistic model, Dirichlet-multinomial mixtures (DMM) [33] which identifies clusters of samples based on bacterial community composition. Based on a Laplace approximation, three distinct sample clusters, termed Dirichlet states (DSI-III) represented the best model fit (Additional file 6: Figure S3A); DSI comprised 26 subjects (*n* = 9/10 healthy, *n* = 17 CRS), DSII comprised 14 CRS patients, and DSIII comprised 28 CRS patients and one healthy subject. Upon chart review, it was noted that this particular healthy subject had allergic rhinitis. DS clusters were confirmed as compositionally distinct by PERMANOVA (weighted UniFrac PERMANOVA; *p* = 0.001, 31.8% variation; Fig. 2a), a finding that was robust irrespective of the distance matrix used to analyze the 16S rRNA data (Table 3, Additional file 6: Figure S2A). Both weighted and unweighted UniFrac distance matrices significantly explained DS-defined sample clustering, indicating that both bacterial phylogeny and rarer taxa in these communities discriminated DS groups. To further confirm this, sequence reads associated with the dominant family in each sample were removed and the data reanalyzed. DS classification remained significantly related to community composition (weighted UniFrac PERMANOVA; *p* = 0.001, 18.2% variation; Additional file 6: figure S2B) indicating that patterns of co-associated lower abundance taxa are discrete and relatively conserved within each of the three DS microbiota.

**Table 3:** Weighted and unweighted UniFrac distance matrix

PERMANOVA (Dirichlet states)	*r* ^2^	*p* value
Weighted UniFrac	0.326	0.001
Unweighted UniFrac	0.182	0.001
Bray-Curtis	0.195	0.001
Canberra	0.099	0.001

The proportion of healthy subjects and CRS patients with or without pulmonary co-morbidities varied significantly across DSI-III (Chi-squared; *p* = 0.0007; Fig. 2b), with DSI possessing the least and DSII the greatest proportion of CRS patients with concomitant lower airway disease (CRS+CF and CRS+A). This implicates a co-association between specific pathogenic sinus community states and lower airway disease and also provides the first evidence that specific pathogenic sinus microbiota are common to both CF and asthmatic patients. While recent antibiotic use trended towards significance, it only explained a very minor portion of community compositional variance (PERMANOVA; *p* = 0.052, *r* ^2^ = 0.036; Table 2) and did not differ across DSI-III (Chi-squared; *p* = 0.149). This is likely because these microbiota exist in antimicrobial resistant biofilms on the sinus mucosal surface [35]. Disease severity, as measured by Lund-Mackay radiographic scores also did not differ across DSI-III (ANOVA *p* = 0.825), suggesting that distinct pathogenic microbiota may drive equally severe disease symptoms, albeit via distinct mechanisms (it should be noted that these patients were undergoing functional endoscopic sinus surgery at the time of sample collection).

DSIII was the largest group and was comprised of patients whose sinus mucosal microbiota represented a compositional continuum dominated by either *Staphylococcaceae* (*Firmicutes*) or *Corynebacteriaceae* (*Actinobacteria*). Since these taxa are phylogenetically distinct, are known competitors in the upper airways [36, 37], and elicit unique immune responses [38], we identified *Corynebacteriaceae-* or *Staphylococcaceae*-dominated patients within this group as distinct DSIII sub-groups, identified as DSIII(a) (*n* = 9) or III(b) (*n* = 19; Fig. 2b), respectively. This sub-grouping strategy was statistically supported by hierarchical clustering analysis on a weighted UniFrac distance matrix (au, *p* < 0.05; Additional file 6: Figure S3B), and we confirmed the existence of a reciprocal relationship between *Corynebacteriaceae* [DSIII(a)] and *Staphylococcaceae* [DSIII(b)] relative abundance across DSIII samples (Additional file 6: Figure S3C).

Each pathogenic microbiota state (DSI-III) was characteristically dominated by a distinct bacterial family that co-associated with a relatively unique suite of lower abundance taxa (Fig. 2c). To identify taxonomic differentials characteristic of each CRS microbiota state, each was compared to healthy subjects using zero-inflated negative binomial (ZINB) regression (Fig. 2d–g, Additional file 3). The identity and magnitude of depleted taxa was relatively consistent irrespective of the CRS microbiota state examined and included *Streptococcus*, *Rothia*, *Haemophilus*, and *Lactobacillales* members (ZINB; *p* < 0.05, *q* < 0.10; Fig. 2d *–*g). The magnitude and types of taxa enriched in CRS patients differed by community state (Fig 2c) and were most pronounced in DSIII(a) and III(b), which exhibited relatively large *Corynebacterium* or *Staphylococcus* enrichments, respectively. DSI, though most compositionally similar to healthy controls, exhibited relative enrichment of *Streptococcus* as well as *Porphyromonas*, *Tannerella*, *Treponema*, *Bacteroides*, *Dialister*, and *Akkermansia* (ZINB; *p* < 0.05, *q* < 0.05)*.* DSII, dominated by *Pseudomonadaceae*, was also relatively enriched for *Fusobacterium*, *Aggregatibacter*, *Achromobacter*, and *Prevotella* (ZINB; *p* < 0.05, *q* < 0.05), known airway pathobionts characteristically enriched in CF and asthmatic lungs [1, 7, 39, 40]. Presumably, this reflects the increased number of such patients in this sub-group and indicates that archetypal lower airway microbiome dysbioses in CF and asthmatic patients may also be reflected in the upper airway bacterial community composition of these patients. While DSIII(a) and III(b) shared substantial taxonomic overlap, explaining their statistical grouping into a single DMM cluster, DSIII(a) was uniquely enriched for *Sphingomonas* (ZINB; *p* < 0.0001, *q* < 0.0001; Fig. 2f) and DSIII(b) uniquely co-enriched for eight taxa absent in III(a) [*Actinobacteria*, *Bifidobacterium*, *Haemophilus*, *Enterobacteriaceae*, *Pseudomonadaceae*, *Sphingomonadaceae* (unclassified genus), *Selenomonas*, and *Streptophyta* (ZINB; *p* < 0.05, *q* < 0.05; Fig. 2g)]*.*

Because the majority of healthy individuals were classified into DSI, we also compared DSII, III(a) and III(b) individually to DSI. General concordance was observed between taxa enriched in DSII or III when compared to either DSI or healthy subjects (ZINB; *p* < 0.05, *q* < 0.05; Additional file 6: Tables S1-S3). The primary discriminating genera for each DS remained consistent; differences were only observed in a select few low abundance taxa, validating the observation that the DSI microbiota was compositionally similar to that of healthy subjects. When compared to DSI, DSII remained enriched for *Aggregatibacter*, *Achromobacter*, *Fusobacterium*, and *Prevotella* but was also relatively enriched for *Pseudomonas* (ZINB; *p* = 0.004, *q* = 0.026). DSIII(a) and III(b) remained highly enriched for *Corynebacterium* or *Staphylococcus* although *Cloacibacterium* were uniquely co-enriched with *Corynebacterium* and *Serratia* were uniquely co-enriched with *Staphylococcus* when these groups were compared with DSI*.*

### Predicted functional capacity discriminates sinus bacterial Dirichlet states

**Fig. 3:**
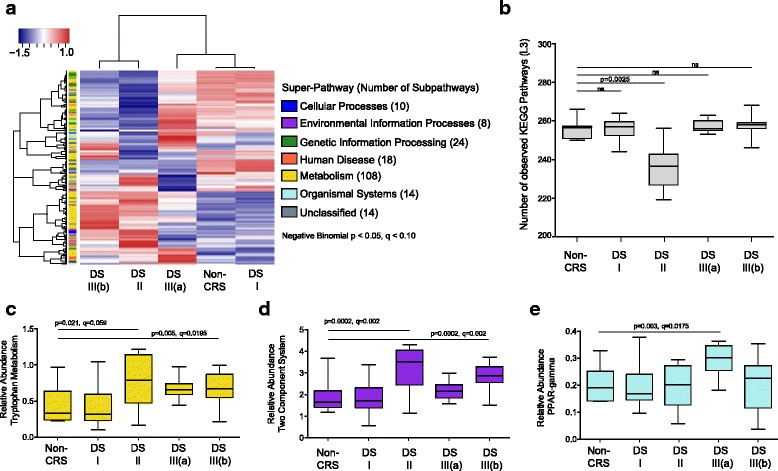
Variation in predicted metagenomes associated with each Dirichlet state. **a** Heatmap of significantly enriched (*red*) or depleted (*blue*) KEGG pathways collapsed at level 3 compared to healthy subjects (negative binomial; *p* < 0.05, *q* < 0.10, DS vs. nonCRS). For visualization, read counts were normalized [log2(*x* + 1)] and scaled by row. **b** DSII is significantly functionally depleted, measured by total unique KEGG pathways compared to healthy individuals (permutational *t* test; *q* = 0.0025). **c** Tryptophan metabolism is enriched in DSII [*Pseudomonadaceae-*defined, (negative binomial; *p* = 0.021, *q* = 0.059)] and DSIII(b) [*Staphylococcaceae-*defined (negative binomial; *p* = 0.005, *q* = 0.012)]. **d** The two-component response system virulence pathway is enriched in DSII [*Pseudomonadaceae-*defined, (negative binomial; *p* = 0.0002, *q* = 0.002)] and DSIII(b) [*Staphylococcaceae-*defined (negative binomial; *p* = 0.0002, *q* = 0.002)]. **e** PPAR-γ signaling pathway is enriched in DSIII(a) [*Corynebacteriaceae-*defined (negative binomial; *p* = 0.003, *q* = 0.0175)]

Bacterial metagenomes were predicted *in silico* for each patient using PICRUSt, an algorithm which uses biomarker gene sequence data i.e., 16S rRNA to infer evolutionarily conserved functional gene capacity using representative sequenced and predicted ancestral genomes. Associated Nearest Sequenced Taxon Index (NTSI) scores which indicate the degree of relatedness between OTUs and sequenced genomes used for PICRUSt predictions are detailed in Additional file 7. Each microbiota state was predicted to encode a distinct metagenome (Bray-Curtis PERMANOVA; *p* = 0.001, 23.2% variation explained; Additional file 6: Figure S4) and a total of 196 KEGG pathways differentiated pathogenic microbiota states compared control patients (three-model test; *p* < 0.05, *q* < 0.10; Fig. 3a). Only 21 KEGG pathways discriminated patients with distinct lower airway co-morbidities (asthma+/−, CF; Kruskal-Wallis; *p* < 0.05, *q* < 0.10; Additional file 6: Table S4), indicating substantial overlap in sinus microbiota function in the upper airways of CRS patients with distinct lower airway diseases. Compared to healthy microbiota, the DSII group was the least functionally diverse (permutational *t* test; *q* < 0.05; Fig. 3b) and depleted of 67 KEGG pathways for lipid, carbohydrate, terpenoid, and xenobiotic metabolism. DSII and III(b) were both significantly enriched in bacterial virulence pathways, including two-component response systems, and for fatty acid and tryptophan metabolism pathways associated with inflammation (negative binomial; *p* < 0.05, *q* < 0.05; Fig. 3c–d, Additional file 6: Table S5), when compared to healthy controls. DSI patients were depleted of polyketide and folate biosynthesis and enriched for a pathway responsible for ansamycin biosynthesis, a microbial secondary metabolite with a broad range antimicrobial activity (poison; *p* < 0.0001, *q* < 0.0001; Additional file 6: Table S5A) [41]. *Corynebacterium*-dominated DSIII(a) was characterized by both peroxisome proliferator-activated receptor-γ [(PPAR-γ) negative binomial; *p* = 0.003, *q* = 0.018; Fig. 3e, Additional file 6: Table S5C] and the retinoic acid-inducible gene-1 (RIG-I) signaling pathways (negative binomial; *p* = 0.015, *q* = 0.062; Additional file 6: Table S5C), both of which have been shown to be increased in eosinophilic polyp tissue in CRS patients [42, 43].

### Sinus microbial communities correlate with clinical outcomes

**Fig. 4:**
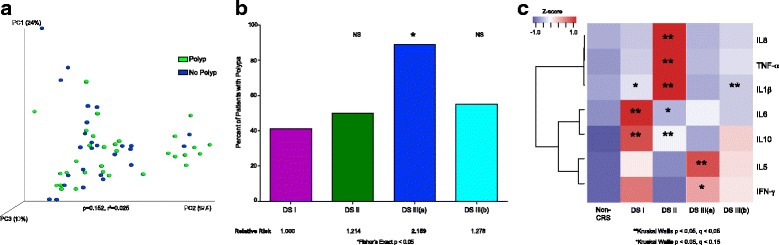
Microbial states confer a differential risk for polyposis and are significantly associated with distinct profiles of host immune response. **a** Multivariate permutation (PERMANOVA) testing of presence or absence of polyps at time of surgery does not indicate a significant relationship at the whole-community level (*p* = 0.152, 2.5% variation explained). **b** Patients with DSIII(a) have a significantly increased risk for polyposis (88.9% of patients have polyps; Fisher’s exact; *p* = 0.032, relative risk = 2.159 compared to DSI). **c** Heatmap of Z score-normalized mean fold change (2^−∆∆Ct^) for each gene examined indicates that immune responses distinct from that of healthy subjects are evident in CRS patients; samples are grouped by DS and healthy individuals (*indicates Kruskal-Wallis; *p* < 0.05, *q* < 0.15; **indicates Kruskal-Wallis; *p* < 0.05, *q* < 0.05; DS vs. nonCRS)

Bacterial community composition within the cohort did not correlate with polyposis (weighted UniFrac; PERMANOVA; *p* = 0.152, 2.5% variation; Fig 4a). However, based on the microbiota and predicted metagenome data, specifically, the enrichment of RIG-I and PPAR-γ signaling pathways (previously associated with polyposis) in the *Corynebacterium*-dominated DSIII(a) patients, we predicted that these patients would exhibit significantly increased incidence of polyposis. We therefore performed an assessment of polyp relative risk across microbiologically discrete CRS patient sub-groups compared to DSII patients, who possessed compositionally similar microbiota to those of healthy subjects and the lowest incidence of polyposis (41%; 7 of 17 patients). As expected, the DSIII(a) sub-group exhibited a significantly higher relative risk of polyposis compared to all of the other microbiologically defined patient sub-groups, with 89% (8 of 9 patients) of patients in this group exhibiting polyposis (Fisher’s exact; relative risk = 2.159, *p* = 0.039; Fig 4b).

### Bacterial co-colonization patterns correlate with patterns of host gene expression

To determine, as we hypothesized, whether distinct DS-induced discrete host immune responses, we used qPCR to measure expression of innate and adaptive genes previously associated with CRS (Th1, Th2, Th17 and Treg cytokines, mucin, and epithelial barrier genes) using RNA extracted in parallel with DNA used to profile microbial communities from sinus brushings of all subjects. Fold change in gene expression (compared to healthy subjects) was used to generate a multivariate immune response profile for each subject.

Each CRS DS group exhibited a significantly distinct host immune response, the specifics of which varied across CRS patient sub-groups (Fig. 4c; full array in Additional file 6: Figure S5). DSI, II, and III(b) patients exhibited significantly increased *IL-1β*, implicating macrophage and inflammasome involvement in these patients. In addition to *IL-1β*, patients in DSII also exhibited increased *IL-6*, *TNF-α*, *IL-8*, and *IL-10* expression (Kruskal-Wallis; *p* < 0.05, *q* < 0.05), suggestive of an epithelial/endothelial and/or macrophage-driven mucosal inflammatory response. DSI patients, whose microbiota composition differed subtly in taxonomic content from healthy individuals, were immunologically distinct and exhibited significantly increased *IL-1β*, *IL-6*, and *IL-10* compared to healthy individuals (Kruskal-Wallis; *p* < 0.05, *q* < 0.05). Thus, subtle taxonomic differences may influence the activity of this microbiota, or, alternatively, non-bacterial microbiota members may contribute to immune-stimulation in this subset of patients. DSIII(a) patients who are at higher relative risk for polyposis and whose sinus mucosal microbiome was enriched for *Corynebacterium* and predicted to encode PPAR-γ and RIG-I signaling pathways were the only group to exhibit a significant increase in *IL-5* expression (Kruskal-Wallis; *p* < 0.05, *q* < 0.05; Fig 4d). *IL-5* is a potent activator of eosinophils, the dominant immune cell type in polyp tissue in Western populations of CRS patients [44]. Furthermore, these patients also had increased levels of *IFN-γ* (Kruskal-Wallis; *p* = 0.017, *q* = 0.107), which has been associated with non-eosinophilic polyposis [45]. Collectively, these findings indicate that distinct dysbiotic pathogenic bacterial microbiota states exist in CRS patient sub-groups that differ in relative risk for polyposis and induce discrete immune responses related to their clinical phenotypes.

## Discussion

Clinical diagnosis of CRS is somewhat subjective and often does not correlate well with patient outcomes [19]. Improved stratification of patients offers the opportunity to better tailor therapeutic regimens and advance towards the ultimate goal of personalized therapy. A previous study of the CRS-associated microbiota demonstrated evidence for mucosal microbiota collapse in patients with severe disease and enrichment of *C. tuberculostearicum* [5]*.* That study also noted that though the number of CRS patients was very small, they parsed into two distinct groups based on sinus microbiota composition. In the current study, we validate previous findings and extend them, demonstrating that the CRS bacterial microbiota can exist in at least four distinct taxonomic states (one of which is dominated by *Corynebacteriaceae*). We suspect that these represent a gradient of pathogenic microbial co-colonizations that are related to patient treatment history and/or disease progression. Previous CRS microbiota studies have described high inter-patient taxonomic variability and dominance of common respiratory pathogens *Corynebacterium*, *Staphylococcus*, *Pseudomonas*, and anaerobes such as *Fusobacterium* and members of *Prevotellaceae* [11–13, 46, 47]. These genera also feature prominently in our study, but we expand upon these findings to demonstrate that these respiratory pathogens co-associate with distinct and reproducible microbial partners and explain a large proportion of the observed inter-personal microbiota variation in CRS patients. These microbiologically distinct states are predicted to encode different metagenomes, are associated with a characteristic innate and adaptive host immune response, and differ significantly in the incidence of nasal polyposis, an important clinical phenotype of CRS.

Approximately one fifth of CRS patients had a mucosal microbiota characteristically enriched for *Corynebacteriaceae* and depleted of *Streptococcus.* Lemon and colleagues recently demonstrated that *Corynebacterium accolens*, a common skin commensal, metabolizes triacylglycerols in nasal secretions to oleic and linoleic acid, which inhibits *Streptococcus pneumoniae* growth [48]. Metagenome predictions indicated that the *Corynebacteriaceae* OTUs in dysbiotic CRS patient microbiota also encode the capacity for linoleic acid biosynthesis, suggesting that this mechanism of *Streptococcus* inhibition may play a role in deterministically shaping the pattern of co-colonizing species around this dominant respiratory pathogen in the chronically inflamed sinus microbiota. However, further in vitro and in vivo studies are required to determine whether this mechanism plays a role in defining CRS microbiota composition. Independent of this pathway, a recently described phylogenetic-related species, *Corynbecterium pyruviciproducens*, has been shown to stimulate dendritic cell maturation and proliferation and up-regulate Th2 responses in mice [49]. Additionally, the lipoarabinomannan-based lipoglycans of *Corynbecterium glutamicum* induce Th17 responses via TLR2 recognition on dendritic cells [50], indicating several discrete pathogenic pathways exist in this genus. In our study, *Corynebacteriaceae-*defined microbial communities were enriched in PPAR-γ and RIG-I pathways. PPAR-γ, a lipid-sensing receptor, controls gene expression and metabolism and has recently been shown to regulate eosinophil activation in polyp tissue of CRS patients [42]. It has also been associated with asthma [51] and airway remodeling following allergic inflammation in mice [52]. RIG-I, an intracellular sensor of viral DNA, is elevated in nasal polyp tissue [43] and is induced by *IFN-γ* [53]. Consistent with these observations, patients possessing a *Corynebacteriaceae*-dominated community state were uniquely associated with increased *IL-5* and *IFN-γ* gene expression and were at a higher risk for developing polyposis. Mounting evidence suggests that members of this family, particularly in the context of a taxonomically and functionally depleted sinus microbiota, represent a group of underappreciated pathobionts, whose activities induce T_H_2-skewed immune responses.

Of the remaining DMM-identified microbial states, patients classified into DSII (*Pseudomonadaceae*-dominated) were the least functionally diverse, the most immunologically active, and housed the greatest proportion of CF and asthma patients, who also commonly exhibit lower airway microbiota dominated by this family. Predicted functional enrichments in DSII included pathways involved in tryptophan metabolism and lipopolysaccharide biosynthesis, both of which induce host inflammatory responses [54]. For example, recent studies have demonstrated that HIV-infected patients with the greatest degree of peripheral immune activation are enriched for *Pseudomonas* species in their gastrointestinal microbiota and that isolates of *Pseudomonas* from these patients exhibit the capacity to catabolize tryptophan to pro-inflammatory kynurenine in vitro [55, 56]. Interestingly, other co-colonizing members of the DSII community are known producers of tryptophan e.g., *Achromobacter* [57], implicating metabolic cross-feeding between the dominant respiratory pathogen and it's co-colonizers as a deterministic mechanism that plausibly underlies their frequent co-association in *Pseudomonas*-dominated sinus microbiota. Additionally, tryptophan metabolites increase biofilm formation [58, 59] and virulence gene expression [60, 61], indicating that enhanced capacity for the production and metabolism of this crucial amino acid by co-associated members of this community state may be critical to enhanced antimicrobial resistance and pathogenicity. Immunologically, patients in DSII, which had the highest prevalence of CRS+CF patients, exhibited increases in genes associated with neutrophil and macrophage activation, including *TNF-α* and *IL-8*, which is consistent with CF airway immune responses associated with strains of *P. aeruginosa* specifically adapted to the lung environment [62, 63]. *IL-1β* gene expression was increased in DSI, II, and III(b), which may indicate a role for inflammasome activation in CRS patients with T_H_1-skewed disease. Inflammasomes are multi-meric protein complexes that assemble in cells to control the production of *IL-1β* and *IL-18* following activation by pathogen-associated molecular patterns (PAMPs), such as peptidoglycan [64].

The goal of this study was to better understand CRS patient heterogeneity by leveraging high-resolution microbiota profiles to stratify patients into discrete sub-groups and to determine whether such a stratification strategy explained immunological and clinical outcomes in these patients as has been demonstrated in other chronic diseases [4, 22, 23, 65]. We demonstrate the existence of distinct microbiota states and show that they are robust and encode unique functional attributes that correlate with mucosal immune responses and clinical outcomes. We recognize that this cross-sectional study cannot address whether these microbiota states are stable or transient; however, it is plausible that they represent a gradient of pathogenic bacterial community successional states associated with disease progression. It will be interesting to determine whether medical management of CRS, such as antimicrobial treatment or surgery, alters a patient’s microbiota state and associated inflammatory response towards that of a different conformation and whether the states we have identified are in fact related to disease progression or duration. Antibiotics can rapidly and pervasively shift the composition of the microbiota in the human gut [66, 67] and can influence sinus microbial composition, at least in the short term [68]. Future studies will examine the effects of medical and surgical management of CRS on the stability of the disease microbiota. Although concomitant lower airway co-morbidities (asthma or CF) explained a small portion of beta diversity variation, we demonstrate that some asthmatics and cytsic fibrosis patients share the same sinus microbiot a state. This observation suggests that subsets of patients with clinically distinct respiratory diseases share the same pathogenic microbiota and host immune response. The concept that discrete respiratory diseases have overlapping pathophysiology has been recently explored in asthma and chronic obstructive pulmonary disease (COPD) [69, 70]. Our data suggests this phenomenon may extend to CF and be explained, at least in part, by overlapping microbiota colonization states. Furthermore, enrichment of *Proteobacteria* in the lower airways patients with established asthma or CF is well documented [7, 71, 72]; our findings demonstrate that lower airway bacterial biomarkers of these respiratory diseases exist in the upper respiratory tract, which may represent a source of pathogenic microbes for lower airway colonization. Future studies will incorporate more thoroughly characterized asthmatics, since asthma immune subtypes are well described and may plausibly be explained by the microbiota states observed in our study [73].

In this initial study, we did not profile viral or fungal components of these microbiota states, which also likely play a role in driving the observed bacterial heterogeneity or host immune response. We also recognize the limitations of using a predictive algorithm to infer metagenome content, particularly since PICRUSt has been most thoroughly studied in the GI tract, (though PICRUSt predictions on nasal samples from the Human microbime Project have been shown to be robust when compared to shotgun metagenome sequencing [33]). Future studies will use shotgun metagenomics and transcriptomics approaches to confirm PICRUSt-predicted metagenomes as well as to identify viral and fungal taxa in these patients. We anticipate that metagenomics, in parallel with metabolomics and transcriptomics will substantially improve our capacity to meaningfully stratify patients based on their microbiome.

We observed a non-significant trend towards an association between antibiotic usage and microbiota composition in our study. It is possible that this study was not sufficiently powered to find an association between antibiotic use and microbial composition and that the compositional differences between CRS and healthy subjects may, at least in part, be antibiotic-mediated. We are continuing to recruit patients and will examine this possibility in larger cohorts of cases and controls. Finally, though the precise cellular source of the cytokines induced by the pathogenic bacterial community states cannot be gleaned from our gene expression studies of human sinus mucosa, we identified significantly up-regulated genes associated with each state that warrant further investigation. Despite these limitations, the microbial and immunological features described herein provide an explanation for CRS patient heterogeneity and provide a foundation for improved understanding of how distinct pathogenic sinus microbiota may collectively and distinctly drive mucosal disease processes in CRS patients.

## Conclusions

Heterogeneity among CRS patients is poorly understood and represents a significant barrier to disease treatment and to the development of more effective therapies. This study validates and extends previous findings that show collapse of mucosal-associated microbiota in CRS patients [5, 12, 47]. Here, we demonstrate that CRS microbiota can exist in at least four compositional states that are predicted to have distinct functional attributes, correlate with distinct host immune responses, and associate with differential risk for nasal polyps, an important clinical disease phenotype. The presence of *Corynebacteriaceae*-dominant microbial communities in CRS patients were associated with increased *IL-5* gene expression and increased risk for nasal polyps while the remaining three microbial community states were immunologically diverse and were not associated with polyp risk. These findings support prior studies that characterize the immunological heterogeneity of CRS patients using similar clustering approaches [20], but by examining microbial signatures, our studies may provide an explanation for these diverse immune profiles that exist within this patient population. The microbial and immunological features described here may inform strategies for tailored therapy in this patient population.

## Additional files

Additional file 1: Edited pvclust R code for use with a precomputed distance matrix. (R 13 kb)

Additional file 2: Workflow of commands for pvclust. (R 1 kb)

Additional file 3: Source file (1) for pvclust. (R 7 kb)

Additional file 4: Source file (2) for pvclust. (R 13 kb)

Additional file 5: Read me file for pvclust. (TXT 908 bytes)

Additional file 6: Supplemental Information. **Figure S1A.** Lund-MacKay scores associated with disease state. No differences were observed between CRS, CRS+A or CRS+CF patients; B. CRS-CF patients are significantly younger than CRS+A patients (ANOVA, Tukey’s *p* = 0.041); however, no differences in age were observed for pairwise comparisons between the other groups (*p* > 0.05, Tukey’s post hoc test). **Figure S2A.** PCoA of an unweighted UniFrac distance matrix colored by DSI-IIIb and healthy (PERMANOVA *p* = 0.001, 18.2% variation explained); B. PCoA of weighted UniFrac distance matrix after dominant sequence reads associated with the dominant family in each sample were removed demonstrating that DS still significantly explains variation in community composition despite removal of the dominant taxon from each sample (PERMANOVA *p* = 0.001, 17.6% variation explained). **Figure S3A.** Laplace model fit demonstrates three distinct Dirichlet multinomial mixtures groups. B. Hierarchical cluster analysis using a weighted-UniFrac distance matrix showing that microbiomes enriched in *Corynebacteriaceae* forms a distinct cluster (au *p* = 100). Heatmap shows relative abundance of the bacterial general that comprise >90% of the total sequence reads. C. Reciprocal relationship between *Corynebacteriaceae* and *Staphylococcaceae.* **Figure S4.** PICRUSt-predicted functional variation across microbial Dirichlet states shows significant functional differences A. PCoA of Canberra distance matrix; PERMANOVA *p* = 0.001, 21.7% of variation explained) B. PCoA of Bray-Curtis distance matrix; PERMANOVA *p* = 0.001, 22.0% of variation explained). **Figure S5.** Expression levels of all host immune genes measured by QPCR (*indicates Kruskal-Wallis *p* < 0.05, *q* < 0.15; **indicates Kruskal-Wallis *p* < 0.05, *q* < 0.05; DS vs. nonCRS). **Table S1–S5.** (ZIP 1671 kb)

Additional file 7: Nearest Taxon Sequence Index (NTSI) scores for PICRUSt predictions. (XLSX 41 kb)

## Abbreviations

AIC: Akaike information criterion
CF: Cystic fibrosis
CRS: Chronic rhinosinusitis
CT: Computed tomography
DMM: Dirichlet-multinomial mixtures model
DS: Dirichlet state
KEGG: Kyoto Encyclopedia of Genes and Genomes
NTSI: Nearest taxon sequence identity
OTU: Operational taxonomic unit
PCoA: Principal Coordinates Analysis
PERMANOVA: Permutational multivariate analysis of variance
