# Supplementary material for: Compositionally and functionally distinct sinus microbiota in chronic rhinosinusitis patients have immunological and clinically divergent consequences
Source: Microbiome. 2017 May 12;5:53. doi: 10.1186/s40168-017-0266-6 (PMC5427582; doi:10.1186/s40168-017-0266-6)
Supplement: Supplementary file 6 — Supplemental Information. Figure S1A. Lund-MacKay scores associated with disease state. No differences were observed between CRS, CRS+A or CRS+CF patients; B. CRS-CF patients are significantly younger than CRS+A patients (ANOVA, Tukey’s p = 0.041); however, no differences in age were observed for pairwise comparisons between the other groups (p > 0.05, Tukey’s post hoc test). Figure S2A. PCoA of an unweighted UniFrac distance matrix colored by DSI-IIIb and healthy (PERMANOVA p = 0.001, 18.2% variation explained); B. PCoA of weighted UniFrac distance matrix after dominant sequence reads associated with the dominant family in each sample were removed demonstrating that DS still significantly explains variation in community composition despite removal of the dominant taxon from each sample (PERMANOVA p = 0.001, 17.6% variation explained). Figure S3A. Laplace model fit demonstrates three distinct Dirichlet multinomial mixtures groups. B. Hierarchical cluster analysis using a weighted-UniFrac distance matrix showing that microbiomes enriched in Corynebacteriaceae forms a distinct cluster (au p = 100). Heatmap shows relative abundance of the bacterial general that comprise >90% of the total sequence reads. C. Reciprocal relationship between Corynebacteriaceae and Staphylococcaceae. Figure S4. PICRUSt-predicted functional variation across microbial Dirichlet states shows significant functional differences A. PCoA of Canberra distance matrix; PERMANOVA p = 0.001, 21.7% of variation explained) B. PCoA of Bray-Curtis distance matrix; PERMANOVA p = 0.001, 22.0% of variation explained). Figure S5. Expression levels of all host immune genes measured by QPCR (*indicates Kruskal-Wallis p < 0.05, q < 0.15; **indicates Kruskal-Wallis p < 0.05, q < 0.05; DS vs. nonCRS). Table S1–S5. (ZIP 1671 kb) [file 40168_2017_266_MOESM6_ESM.zip › Microbiome_Supplemental_Methods040617_Revised_Clean_SVL.docx]

**Supplemental Materials.**

**Supplementary Figures.**

**Fig. S1A.** Lund MacKay scores associated with disease state. No differences were observed between CF-CRS patients and CRS patients with or without asthma; **B.** CF-CRS patients are significantly younger than non-CF CRS patients with asthma (ANOVA, Tukey’s p=0.041), however, no differences in age were observed for pairwise comparisons between the other groups (p>0.05, Tukey’s *post hoc* test).

**Fig. S2A.** PCoA of an unweighted UniFrac distance matrix colored by DSI-IIIb and healthy (PERMANOVA p=0.001, 18.2% variation explained); **B.** PCoA of weighted UniFrac distance matrix after dominant sequence reads associated with the dominant family in each sample were removed demonstrating that DS still significantly explains variation in community composition despite removal of the dominant taxon from each sample (PERMANOVA p=0.001, 17.6% variation explained).

**Fig. S3A.** Laplace model fit demonstrates three distinct Dirichlet multinomial mixtures groups. **B.** Hierarchical cluster analysis using a weighted UniFrac distance matrix showing that microbiomes enriched in *Corynebacteriaceae* forms a distinct cluster (au p=100). Heatmap shows relative abundance of the bacterial general that comprise >90% of the total sequence reads. **C.** Reciprocal relationship between *Corynebacteriaceae* and *Staphylococcaceae.*

**Fig. S4.** PICRUSt-predicted functional variation across microbial Dirichlet States shows significant functional differences A. PCoA of Canberra distance matrix; PERMANOVA p=0.001, 21.7% of variation explained) B. PCoA of Bray-Curtis distance matrix; PERMANOVA p=0.001, 22.0% of variation explained)

**Fig. S5.** Expression levels of all host immune genes measured by QPCR (* indicates Kruskal Wallis p < 0.05, q < 0.15; ** indicates Kruskal Wallis p < 0.05, q < 0.05; DS vs. non-CRS).

*16S rRNA gene sequence processing.*

Sequence analysis of 16S rRNA data was performed using the QIIME version 1.8.0^1^ and in the R environment. Despite many recent studies analyzing single-end Illumina sequences^2,3^, we selected to assemble paired-end sequences to increase the number of high-quality (>q30) bases in order to capture the full length of the V4 region. The 254bp V4 region of the rRNA gene was sequenced 251bp into Read 1 and 151bp in Read 2 (251x151). We chose to sequence 151 bp into read 2 as a time-saving measure on a highly-used MiSeq instrument. Since, on average, run quality was reduced at the end of the Illumina reads (approximately after 200-220bp per FastQC analysis, data not shown), a reverse read of 151bp was sufficient to provide overlap at the low-quality ends of the read. This approach was sufficient to increase the quality of the 3’ ends of each read and output full-length V4 reads at q>30. Each 251x151 paired read was assembled using FLASh (Fast Length Adjustment of SHort reads^4^) with parameters: -r 251 -f 300 -s 30 -m 15. Assembled sequence data were de-multiplexed by barcode and sequences were quality filtered in QIIME 1.8.0 as follows. Phred quality scores of Q30 were retained; if three consecutive bases were <Q30, then the read was truncated before the low-quality bases. The resulting read was retained in the dataset if it was at least 75% of the original length. Operational taxonomic units (OTUs) were picked at 97% sequence identity using uclust against the Greengenes database (13_5)^5,6^. Reads that failed to hit the reference sequence collection were retained and clustered *de* *novo*. Sequences were aligned using PyNAST and taxonomy was assigned using uclust in the qiime environment^7^. PyNAST-aligned sequences were chimera checked using ChimeraSlayer and putative chimeras were removed from the OTU table. Eight OTUs that were present in the negative extraction controls, which corresponded to members of *Pseudomonadaceae, Delftia, Mycoplana, Bradyrhizobium,* and *Neisseriaceae,* were removed from the OTU table, since these were represented by <100 sequences in the extraction blank and the specific OTUs have been previously identified as contaminants in extraction controls in our lab and in published studies^8^. A phylogenetic tree was then built using FastTree^9^ and used to compute Faith’s Phylogenetic Diversity and UniFrac distances. Since our rarefaction curves approached an asymptote (indicating adequate community coverage) at a sequence depth 10,055 sequences, and all but 5 samples were sequenced at least to this depth, the OTU table was multiple rarefied to 10,055 high-quality, chimera checked sequences per sample for subsequent analyses using a custom script (https://github.com/alifar76/MicroNorm).

*Predicted Metagenomics.*

Metagenome prediction from the closed-reference OTUs (greengenes 13_5) of the multiple rarefied OTU table was performed using the PICRUSt software (<http://picrust.github.io/picrust/>)^10^. QIIME 1.8.0 was used to analyze the predicted metagenomes. A table of KEGG pathways collapsed from KOs to level 3 was used for subsequent analysis. Since the resulting table had a range of count depths for each pathway, the table was rarefied to 2,000,000 KEGG pathway counts per sample prior to computing between-sample distances (Bray Curtis, Canberra) or testing differential abundances of pathways.

*QPCR for bacterial burden and human gene expression.*

QPCR was used to quantify bacterial burden. The universal primers 338F/518-R (338F, 5’- ACTCCTACGGGAGGCAGCAG -3’^11^ and 518R 5’- ATTACCGCGGCTGCTGG -3’) were used to amplify the 196bp region of the V3-V4 rRNA gene for quantification of total 16S rRNA copy number as previously described^12^. Copy number was normalized to host beta-actin (ACTB-F, 5’- AAGATGACCCAGATCATGTTTGAGACC-3’, ACTB-R, 5’- AGCCAGTCCAGACGCAGGAT-3’). Reaction mixtures (20 μl total) contained 10 μl SYBRgreen MM (2X), 1 μM each primer, 20 ng template DNA, and 4 μl water. Reactions were amplified using the QuantStudio 6 (Life Technologies) per the following conditions: 95 °C for 10 min and 40 cycles of 95 °C for 30 sec, 55 °C for 60 sec, and 72 °C for 30 sec. The data acquisition step was set at 55 °C and a disassociation curve was recorded. Standard curves of known 16S rRNA (*Escherichia coli*) or human β-actin gene copy number were used to calculate copy number in test samples^13^.

To determine whether sinonasal microbial community composition correlated with aberrant host immune responses, mucin secretion or epithelial barrier function, a custom QPCR array was developed (SA Biosciences) and used to quantify host gene expression using RNA extracted in parallel from patient sinus brushes. Contaminating DNA was removed using the Genomic Elimination “GE” buffer as indicated in the RT^2^ First Strand Synthesis kit (Qiagen) from 250 ng of total RNA and cDNA was synthesized buffer RE3 with a spike in control P2 per manufacturer instructions. . Resulting cDNA was used in a 10 μl SYBR green reaction with custom primers for each gene of interest on a Life Technologies Quant 6 QPCR instrument. PCR conditions were as follows: One cycle at 95°C for 10 min, 40 cycles of 95°C for 15s and 60°C for 60 seconds, followed by a melt curve. Expression of *Occludin, Claudin 2, MUC5AC, IL-4, IL-5, IL-6, IL-8, IL-25, IL-17A, IL-10, IL-1β, IL-33, CCL11* (eotaxin), *TSLP* (thymic stromal lymphopoietin), *TNF-α, ARG1, TGFβ1* (transforming growth factor, beta 1), *CLCA1* (chloride channel accessory), and *IFN-γ* were normalized to *β-actin* housekeeping gene by the ΔΔCt method ^14^. Fold change is reported as 2^-ΔΔCt^.

**Supplementary Tables.**

**Table S1.**

| **DS I v. DS II** | | | | |
| --- | --- | --- | --- | --- |
| **OTU_IDs** | **ZINB p value** | **ZINB q value** | **Fold Difference** | **Taxonomy** |
| 274754 | 2.05E-05 | 0.0003 | 611.31 | *Enterobacteriaceae; g__; s__* |
| 545299 | 0.0003 | 0.0029 | 450.07 | *Fusobacteriaceae; g__Fusobacterium; s__* |
| 242070 | 0.0035 | 0.0266 | 284.62 | *Pseudomonadaceae; g__Pseudomonas; s__* |
| 68617 | 5.96E-08 | 1.59E-06 | 230.20 | *Alcaligenaceae; g__Achromobacter; s__* |
| 4302571 | 1.24E-125 | 5.95E-123 | 113.05 | *Prevotellaceae; g__Prevotella; s__* |
| 114510 | 6.39E-08 | 1.62E-06 | 78.92 | *Enterobacteriaceae; g__; s__* |
| 4432431 | 0.0058 | 0.0406 | 52.32 | *Pasteurellaceae; g__Aggregatibacter; s__segnis* |
| 1147942 | 7.99E-28 | 1.92E-25 | 39.48 | *Pasteurellaceae; g__Aggregatibacter; s__* |
| 3678349 | 8.79E-05 | 0.0011 | 25.70 | *Streptococcaceae; g__Streptococcus; s__anginosus* |
| 4466150 | 8.82E-07 | 1.84E-05 | 24.72 | *Pasteurellaceae; g__Aggregatibacter; s__segnis* |
| 4426163 | 2.56E-10 | 1.03E-08 | 20.59 | *Prevotellaceae; g__Prevotella; s__* |
| 610111 | 3.37E-20 | 4.05E-18 | 15.48 | *Prevotellaceae; g__Prevotella; s__* |
| 928538 | 0.0013 | 0.0112 | 15.46 | *Staphylococcaceae; g__Staphylococcus; s__* |
| 656881 | 8.31E-05 | 0.0011 | 13.37 | *Enterobacteriaceae; g__; s__* |
| 4448731 | 0.0005 | 0.0052 | 11.14 | *Fusobacteriaceae; g__Fusobacterium; s__* |
| 3385021 | 0.0001 | 0.0017 | 9.92 | *Staphylococcaceae; g__Staphylococcus; s__* |
| 269901 | 0.0017 | 0.0135 | 8.71 | *Pseudomonadaceae; g__; s__* |
| 144814 | 1.68E-06 | 3.37E-05 | 7.42 | *Enterobacteriaceae* |
| 91557 | 2.08E-09 | 6.66E-08 | 7.23 | *Enterobacteriaceae* |
| 4415943 | 0.0004 | 0.0044 | 6.93 | *Fusobacteriaceae; g__Fusobacterium; s__* |
| 141145 | 0.0046 | 0.0337 | 4.96 | *Enterobacteriaceae; g__; s__* |
| 996487 | 2.83E-07 | 6.80E-06 | 4.07 | *Staphylococcaceae; g__Staphylococcus; s__epidermidis* |
| 939252 | 3.89E-06 | 7.26E-05 | 3.98 | *Staphylococcaceae; g__Staphylococcus; s__* |
| 122049 | 4.04E-08 | 1.14E-06 | 3.96 | *Enterobacteriaceae; g__; s__* |
| 137056 | 2.04E-09 | 6.66E-08 | 3.70 | *Planococcaceae; g__; s__* |
| 4312969 | 0.0044 | 0.0327 | 2.68 | *Staphylococcaceae; g__Staphylococcus* |
| 1076316 | 0.0009 | 0.0084 | 2.53 | *Staphylococcaceae; g__Staphylococcus; s__* |
| New.ReferenceOTU160 | 0.0016 | 0.0130 | 1.75 | *Pseudomonadaceae; g__Pseudomonas; s__* |
| 960695 | 0.0014 | 0.0122 | 1.14 | *Planococcaceae; g__; s__* |
| 4466659 | 7.89E-05 | 0.0010 | 1.03 | *Fusobacteriaceae; g__Fusobacterium; s__* |
| 4415319 | 0.0071 | 0.0485 | 0.96 | *Alcaligenaceae; g__Achromobacter; s__* |
| 1055132 | 0.0073 | 0.0491 | 0.71 | *Staphylococcaceae; g__Staphylococcus* |
| 982266 | 0.0030 | 0.0231 | -0.55 | *[Chromatiaceae]* |
| 4322739 | 0.0003 | 0.0035 | -0.73 | *Dermacoccaceae; g__Dermacoccus; s__* |
| 159017 | 0.0006 | 0.0056 | -0.97 | *Caulobacteraceae; g__; s__* |
| 109060 | 0.0053 | 0.0372 | -1.70 | *Comamonadaceae; g__Delftia; s__* |
| 809192 | 0.0001 | 0.0013 | -2.31 | *Dermabacteraceae; g__Brachybacterium; s__conglomeratum* |
| 4328567 | 0.0032 | 0.0243 | -2.43 | *Comamonadaceae; g__Delftia; s__* |
| 4449609 | 2.03E-05 | 0.0003 | -3.62 | *Sphingomonadaceae; g__Sphingomonas; s__* |
| 4323897 | 0.0028 | 0.0219 | -3.78 | *Oxalobacteraceae; g__; s__* |
| 823916 | 0.0048 | 0.0342 | -5.49 | *Moraxellaceae; g__Enhydrobacter; s__* |
| 142419 | 7.70E-06 | 0.0001 | -5.52 | *Pseudomonadaceae* |
| 4363066 | 0.0002 | 0.0023 | -5.70 | *Pasteurellaceae; g__Aggregatibacter; s__* |
| 668514 | 0.0006 | 0.0056 | -5.99 | *Enterobacteriaceae; g__; s__* |
| 400315 | 3.92E-06 | 7.26E-05 | -6.18 | *Pseudomonadaceae; g__Pseudomonas; s__* |
| 1082607 | 8.36E-21 | 1.34E-18 | -9.27 | *Corynebacteriaceae; g__Corynebacterium; s__* |
| 4331815 | 0.0011 | 0.0097 | -9.98 | *Sphingomonadaceae; g__; s__* |
| 4344371 | 4.68E-05 | 0.0007 | -12.47 | *Sphingomonadaceae; g__Sphingomonas; s__* |
| 4456891 | 9.54E-06 | 0.0002 | -12.93 | *Pseudomonadaceae; g__Pseudomonas; s__* |
| 615020 | 0.0004 | 0.0037 | -13.27 | *Mycoplasmataceae; g__Mycoplasma; s__* |
| 2685602 | 1.21E-05 | 0.0002 | -13.74 | *Comamonadaceae; g__Delftia; s__* |
| 3384047 | 4.31E-05 | 0.0006 | -14.12 | *Streptococcaceae; g__Streptococcus; s__* |
| 610486 | 0.0001 | 0.0017 | -21.62 | *Comamonadaceae* |
| 1053321 | 0.0006 | 0.0055 | -35.16 | *Moraxellaceae; g__Moraxella; s__* |
| 1981302 | 3.44E-10 | 1.27E-08 | -37.45 | *Burkholderiaceae; g__Burkholderia; s__* |
| 254888 | 6.48E-05 | 0.0009 | -41.40 | *Comamonadaceae; g__; s__* |
| 1566691 | 3.32E-07 | 7.58E-06 | -47.23 | *Pseudomonadaceae; g__Pseudomonas; s__* |
| 4416763 | 0.0003 | 0.0035 | -52.71 | *Streptococcaceae; g__Streptococcus; s__* |
| 866280 | 4.48E-06 | 7.98E-05 | -55.89 | *Micrococcaceae; g__Rothia; s__mucilaginosa* |
| 494906 | 0.0002 | 0.0019 | -59.76 | *[Tissierellaceae]; g__Peptoniphilus; s__* |
| 4458959 | 1.97E-11 | 9.45E-10 | -71.25 | *Veillonellaceae; g__Veillonella; s__parvula* |
| 4405869 | 3.47E-07 | 7.58E-06 | -85.44 | *Fusobacteriaceae; g__Fusobacterium; s__* |
| 937813 | 5.73E-05 | 0.0008 | -89.12 | *[Tissierellaceae]; g__Anaerococcus; s__* |
| 4446902 | 4.15E-11 | 1.82E-09 | -115.01 | *Gemellaceae; g__; s__* |
| 4411138 | 9.82E-09 | 2.95E-07 | -121.47 | *Micrococcaceae; g__Rothia; s__mucilaginosa* |
| 495067 | 0.0017 | 0.0140 | -136.15 | *Corynebacteriaceae; g__Corynebacterium; s__* |
| 12574 | 1.19E-14 | 8.19E-13 | -144.40 | *Actinomycetaceae; g__Actinomyces; s__* |
| 4465561 | 2.66E-13 | 1.60E-11 | -233.97 | *Prevotellaceae; g__Prevotella; s__melaninogenica* |
| 4439603 | 3.85E-17 | 3.09E-15 | -234.40 | *Streptococcaceae; g__Streptococcus; s__* |
| 4425214 | 2.39E-12 | 1.28E-10 | -298.58 | *Streptococcaceae; g__Streptococcus; s__* |
| 4309301 | 8.28E-18 | 7.96E-16 | -837.31 | *Streptococcaceae; g__Streptococcus; s__* |

**Table S2.**

| **DS I v. DS IIIa** | | | | |
| --- | --- | --- | --- | --- |
| **OTU_IDs** | **ZINB p value** | **ZINB q value** | **Fold Difference** | **Taxonomy** |
| 1015518 | 4.51E-07 | 2.02E-05 | 2818.55 | *Corynebacteriaceae; g__Corynebacterium; s__* |
| 1062051 | 3.33E-13 | 5.48E-11 | 694.68 | *Corynebacteriaceae; g__Corynebacterium; s__* |
| 4154872 | 3.41E-78 | 1.68E-75 | 102.76 | *[Weeksellaceae]; g__Cloacibacterium; s__* |
| 504674 | 0.0055 | 0.0450 | 54.20 | *[Tissierellaceae]; g__Anaerococcus; s__* |
| 71872 | 0.0018 | 0.0180 | 35.38 | *Comamonadaceae; g__Comamonas; s__* |
| 1116384 | 6.57E-07 | 2.50E-05 | 30.62 | *Comamonadaceae; g__; s__* |
| 1077373 | 3.49E-08 | 2.16E-06 | 28.27 | *Prevotellaceae; g__Prevotella; s__* |
| 3393186 | 2.13E-07 | 1.17E-05 | 20.06 | *Neisseriaceae; g__; s__* |
| 102915 | 4.97E-09 | 4.09E-07 | 19.32 | *Sphingomonadaceae; g__Sphingomonas; s__* |
| 207936 | 1.82E-06 | 5.62E-05 | 17.16 | *[Tissierellaceae]; g__Anaerococcus; s__* |
| 259272 | 0.0008 | 0.0100 | 8.27 | *Bradyrhizobiaceae; g__; s__* |
| 410908 | 6.60E-09 | 4.66E-07 | 8.05 | *Corynebacteriaceae; g__Corynebacterium; s__* |
| 441265 | 1.85E-05 | 0.0004 | 6.09 | *Corynebacteriaceae; g__Corynebacterium; s__* |
| 4312969 | 0.0019 | 0.0188 | 4.04 | *Staphylococcaceae; g__Staphylococcus* |
| 4396717 | 0.0002 | 0.0027 | 4.02 | *Methylobacteriaceae; g__Methylobacterium; s__* |
| 802064 | 0.0025 | 0.0234 | 3.35 | *Burkholderiaceae; g__Burkholderia; s__* |
| 4383166 | 0.0010 | 0.0113 | 3.06 | *Comamonadaceae* |
| 122049 | 0.0056 | 0.0450 | 2.78 | *Enterobacteriaceae; g__; s__* |
| 1068955 | 0.0012 | 0.0125 | 1.31 | *Staphylococcaceae; g__Staphylococcus; s__* |
| 979261 | 0.0027 | 0.0242 | 0.92 | *Staphylococcaceae; g__Staphylococcus; s__aureus* |
| 91557 | 0.0017 | 0.0172 | 0.83 | *Enterobacteriaceae* |
| 982266 | 0.0058 | 0.0461 | -0.27 | *[Chromatiaceae]* |
| 159017 | 0.0002 | 0.0039 | -0.72 | *Caulobacteraceae; g__; s__* |
| 4423410 | 0.0015 | 0.0152 | -0.98 | *Sphingomonadaceae; g__; s__* |
| New.ReferenceOTU32 | 2.45E-11 | 2.42E-09 | -1.28 | *Unassigned* |
| 1049188 | 0.0014 | 0.0150 | -1.48 | *Corynebacteriaceae; g__Corynebacterium; s__* |
| 1100972 | 9.52E-12 | 1.18E-09 | -2.10 | *Streptococcaceae; g__Lactococcus; s__* |
| 4337755 | 0.0027 | 0.0239 | -2.98 | *Gemellaceae; g__; s__* |
| 4449609 | 3.34E-07 | 1.65E-05 | -3.49 | *Sphingomonadaceae; g__Sphingomonas; s__* |
| 4437024 | 0.0005 | 0.0066 | -3.53 | *Streptococcaceae; g__Streptococcus; s__* |
| 1927234 | 0.0002 | 0.0039 | -3.71 | *Leptotrichiaceae; g__Leptotrichia; s__* |
| 511378 | 0.0031 | 0.0261 | -4.17 | *Veillonellaceae; g__Megasphaera; s__* |
| 790466 | 4.43E-06 | 0.0001 | -4.86 | *[Mogibacteriaceae]; g__Anaerovorax; s__* |
| 4302049 | 0.0004 | 0.0048 | -4.89 | *Streptococcaceae; g__Streptococcus; s__* |
| 1029036 | 0.0005 | 0.0064 | -7.02 | *Porphyromonadaceae; g__Porphyromonas; s__* |
| 611110 | 1.43E-30 | 3.52E-28 | -10.13 | *Prevotellaceae; g__Prevotella; s__intermedia* |
| 4424239 | 0.0006 | 0.0074 | -11.98 | *Streptococcaceae; g__Streptococcus; s__* |
| 4431355 | 0.0003 | 0.0042 | -14.05 | *Neisseriaceae; g__; s__* |
| 4456889 | 1.73E-05 | 0.0004 | -15.32 | *Pseudomonadaceae; g__Pseudomonas; s__* |
| 1042479 | 0.0003 | 0.0042 | -16.17 | *Prevotellaceae; g__Prevotella; s__melaninogenica* |
| 4306048 | 3.08E-06 | 8.96E-05 | -16.41 | *Streptococcaceae; g__Streptococcus; s__* |
| 4432431 | 5.09E-05 | 0.0010 | -17.94 | *Pasteurellaceae; g__Aggregatibacter; s__segnis* |
| 4455767 | 8.38E-05 | 0.0016 | -18.52 | *Streptococcaceae; g__Streptococcus; s__* |
| 4296424 | 5.51E-06 | 0.0001 | -28.75 | *Actinomycetaceae; g__Actinomyces; s__* |
| 1053321 | 0.0026 | 0.0239 | -35.13 | *Moraxellaceae; g__Moraxella; s__* |
| 4469359 | 0.0011 | 0.0120 | -40.45 | *Pasteurellaceae; g__Haemophilus; s__* |
| 851704 | 0.0011 | 0.0125 | -43.48 | *[Tissierellaceae]; g__Parvimonas; s__* |
| 4319899 | 0.0003 | 0.0042 | -67.36 | *Fusobacteriaceae; g__Fusobacterium; s__* |
| 4405869 | 0.0025 | 0.0237 | -83.25 | *Fusobacteriaceae; g__Fusobacterium; s__* |
| 4446902 | 8.19E-06 | 0.0002 | -111.88 | *Gemellaceae; g__; s__* |
| 12574 | 1.14E-05 | 0.0003 | -140.94 | *Actinomycetaceae; g__Actinomyces; s__* |
| 4453501 | 6.10E-07 | 2.50E-05 | -152.38 | *Veillonellaceae; g__Veillonella; s__dispar* |
| 1059655 | 8.60E-07 | 3.03E-05 | -219.71 | *Streptococcaceae; g__Streptococcus; s__* |
| 4439603 | 4.65E-05 | 0.0010 | -221.22 | *Streptococcaceae; g__Streptococcus; s__* |
| 4465561 | 9.67E-05 | 0.0018 | -223.64 | *Prevotellaceae; g__Prevotella; s__melaninogenica* |
| 4323555 | 0.0003 | 0.0042 | -234.71 | *Fusobacteriaceae; g__Fusobacterium; s__* |
| 4425214 | 1.14E-06 | 3.75E-05 | -290.55 | *Streptococcaceae; g__Streptococcus; s__* |
| 225088 | 0.0001 | 0.0019 | -297.29 | *Pseudomonadaceae; g__Pseudomonas; s__* |
| 4471251 | 0.0029 | 0.0248 | -306.95 | *Pasteurellaceae; g__Haemophilus; s__* |
| 4477696 | 0.0011 | 0.0120 | -363.78 | *Pasteurellaceae; g__Haemophilus* |
| 22951 | 0.0029 | 0.0249 | -373.26 | *Prevotellaceae; g__Prevotella; s__* |
| 4309301 | 0.0004 | 0.0048 | -796.88 | *Streptococcaceae; g__Streptococcus; s__* |

**Table S3.**

| **DS I v. DS IIIa** | | | | |
| --- | --- | --- | --- | --- |
| **OTU_IDs** | **ZINB P value** | **ZINB q value** | **Fold Difference** | **Taxonomy** |
| 4345285 | 6.02E-05 | 0.0006 | 2119.55 | *Staphylococcaceae; g__Staphylococcus; s__* |
| 4416113 | 7.57E-09 | 2.84E-07 | 433.99 | *Enterobacteriaceae; g__Serratia; s__marcescens* |
| 254888 | 0.0073 | 0.0415 | 139.22 | *Comamonadaceae; g__; s__* |
| 553611 | 7.83E-14 | 5.89E-12 | 125.39 | *Bifidobacteriaceae; g__Bifidobacterium; s__* |
| 4428313 | 9.23E-07 | 2.17E-05 | 113.05 | *Lactobacillaceae; g__Lactobacillus; s__* |
| 4361528 | 0.0010 | 0.0074 | 110.53 | *Moraxellaceae; g__Acinetobacter; s__* |
| 4386317 | 2.49E-23 | 3.28E-21 | 90.41 | *; g__; s__* |
| 4319936 | 3.29E-07 | 8.25E-06 | 65.67 | *; g__; s__* |
| 274365 | 0.0001 | 0.0010 | 48.85 | *Enterobacteriaceae; g__; s__* |
| 928538 | 1.15E-05 | 0.0002 | 41.72 | *Staphylococcaceae; g__Staphylococcus; s__* |
| 656881 | 9.47E-07 | 2.17E-05 | 38.47 | *Enterobacteriaceae; g__; s__* |
| 4415684 | 0.0063 | 0.0359 | 30.22 | *Micrococcaceae; g__Kocuria; s__rhizophila* |
| 4396025 | 1.07E-49 | 2.82E-47 | 24.46 | *Sphingomonadaceae; g__; s__* |
| 1040220 | 0.0024 | 0.0149 | 20.36 | *Staphylococcaceae; g__Staphylococcus* |
| 825808 | 7.08E-06 | 0.0001 | 20.24 | *Bifidobacteriaceae; g__Bifidobacterium; s__* |
| 584109 | 4.38E-91 | 2.30E-88 | 19.84 | *Streptococcaceae; g__Streptococcus; s__* |
| 240252 | 0.0012 | 0.0087 | 18.40 | *Acetobacteraceae; g__Acidocella; s__* |
| 4396717 | 7.06E-09 | 2.84E-07 | 18.03 | *Methylobacteriaceae; g__Methylobacterium; s__* |
| 526682 | 0.0032 | 0.0191 | 14.41 | *Actinomycetaceae; g__Actinomyces; s__* |
| 3385021 | 0.0020 | 0.0130 | 12.77 | *Staphylococcaceae; g__Staphylococcus; s__* |
| New.ReferenceOTU66 | 9.21E-12 | 6.05E-10 | 11.71 | *Actinomycetaceae; g__Actinomyces; s__* |
| 4349519 | 9.49E-28 | 1.66E-25 | 11.57 | *[Tissierellaceae]; g__Anaerococcus; s__* |
| 244657 | 2.72E-05 | 0.0003 | 11.53 | *Bradyrhizobiaceae* |
| 119663 | 1.02E-05 | 0.0001 | 11.22 | *Alcaligenaceae* |
| 1010113 | 2.27E-09 | 1.19E-07 | 10.78 | *Enterobacteriaceae; g__; s__* |
| 139289 | 1.53E-08 | 5.03E-07 | 10.18 | *Pseudomonadaceae; g__Pseudomonas; s__* |
| 326163 | 2.15E-06 | 4.34E-05 | 10.12 | *Thermaceae; g__Meiothermus; s__* |
| 509021 | 0.0002 | 0.0021 | 9.51 | *Sphingomonadaceae* |
| 114510 | 0.0014 | 0.0096 | 8.50 | *Enterobacteriaceae; g__; s__* |
| 4423410 | 5.22E-20 | 5.49E-18 | 8.40 | *Sphingomonadaceae; g__; s__* |
| 809192 | 2.99E-16 | 2.62E-14 | 8.30 | *Dermabacteraceae; g__Brachybacterium; s__conglomeratum* |
| 996487 | 0.0001 | 0.0011 | 8.05 | *Staphylococcaceae; g__Staphylococcus; s__epidermidis* |
| 1076316 | 5.98E-06 | 9.05E-05 | 7.67 | *Staphylococcaceae; g__Staphylococcus; s__* |
| 137056 | 1.86E-05 | 0.0002 | 7.40 | *Planococcaceae; g__; s__* |
| 219151 | 8.22E-06 | 0.0001 | 7.12 | *Moraxellaceae; g__Acinetobacter; s__* |
| 939252 | 3.02E-07 | 7.95E-06 | 6.61 | *Staphylococcaceae; g__Staphylococcus; s__* |
| 4421747 | 0.0022 | 0.0138 | 6.49 | *Burkholderiaceae; g__Burkholderia; s__* |
| 258707 | 0.0048 | 0.0280 | 6.28 | *Methylobacteriaceae; g__Methylobacterium; s__* |
| 4312969 | 8.66E-05 | 0.0009 | 5.78 | *Staphylococcaceae; g__Staphylococcus* |
| 4459414 | 2.02E-05 | 0.0002 | 5.28 | *Veillonellaceae; g__Selenomonas; s__noxia* |
| 2468881 | 0.0001 | 0.0010 | 5.24 | *Pseudomonadaceae; g__Pseudomonas; s__* |
| 4374322 | 2.69E-09 | 1.28E-07 | 4.79 | *Moraxellaceae; g__Acinetobacter; s__* |
| 141145 | 0.0016 | 0.0108 | 4.70 | *Enterobacteriaceae; g__; s__* |
| 1116384 | 0.0013 | 0.0087 | 3.95 | *Comamonadaceae; g__; s__* |
| 4473295 | 0.0021 | 0.0136 | 3.63 | *Fusobacteriaceae; g__Fusobacterium; s__* |
| 4383166 | 9.02E-05 | 0.0009 | 3.59 | *Comamonadaceae* |
| 1068955 | 5.85E-06 | 9.05E-05 | 3.28 | *Staphylococcaceae; g__Staphylococcus; s__* |
| 122049 | 0.0079 | 0.0443 | 3.15 | *Enterobacteriaceae; g__; s__* |
| 1111636 | 0.0003 | 0.0022 | 3.09 | *Comamonadaceae* |
| 268968 | 2.70E-08 | 8.36E-07 | 3.09 | *Alcaligenaceae; g__Achromobacter; s__* |
| 678813 | 1.35E-05 | 0.0002 | 3.07 | *Xanthomonadaceae; g__; s__* |
| 368134 | 0.0083 | 0.0459 | 3.07 | *Planococcaceae; g__; s__* |
| 1058950 | 0.0032 | 0.0191 | 2.71 | *Planococcaceae; g__; s__* |
| 960695 | 1.46E-05 | 0.0002 | 2.40 | *Planococcaceae; g__; s__* |
| 544841 | 1.98E-06 | 4.33E-05 | 1.54 | *Sphingomonadaceae; g__; s__* |
| 4306773 | 0.0013 | 0.0089 | 1.50 | *Leptotrichiaceae; g__Leptotrichia; s__* |
| 979261 | 0.0006 | 0.0044 | 1.19 | *Staphylococcaceae; g__Staphylococcus; s__aureus* |
| 984924 | 0.0050 | 0.0290 | 0.99 | *Staphylococcaceae; g__Staphylococcus* |
| 4322998 | 0.0020 | 0.0131 | -0.95 | *Fusobacteriaceae; g__Fusobacterium; s__* |
| 4383953 | 6.02E-06 | 9.05E-05 | -1.31 | *Clostridiaceae; g__; s__* |
| 4428042 | 0.0006 | 0.0046 | -2.20 | *Streptococcaceae; g__Streptococcus; s__* |
| 3678349 | 0.0016 | 0.0108 | -2.68 | *Streptococcaceae; g__Streptococcus; s__anginosus* |
| 4460509 | 1.13E-08 | 3.97E-07 | -2.86 | *Dethiosulfovibrionaceae; g__TG5; s__* |
| 4337755 | 0.0001 | 0.0010 | -2.92 | *Gemellaceae; g__; s__* |
| 4404577 | 0.0007 | 0.0053 | -3.06 | *Peptostreptococcaceae; g__Peptostreptococcus* |
| 1049188 | 2.07E-05 | 0.0002 | -3.83 | *Corynebacteriaceae; g__Corynebacterium; s__* |
| 109413 | 0.0005 | 0.0042 | -3.99 | *Pasteurellaceae; g__Haemophilus; s__parainfluenzae* |
| 1079708 | 4.78E-06 | 8.11E-05 | -4.13 | *Streptococcaceae; g__Streptococcus; s__* |
| 4302049 | 0.0030 | 0.0181 | -4.46 | *Streptococcaceae; g__Streptococcus; s__* |
| 526804 | 0.0004 | 0.0029 | -5.37 | *Streptococcaceae; g__Streptococcus; s__* |
| 513646 | 0.0032 | 0.0191 | -5.83 | *Streptococcaceae; g__Streptococcus; s__* |
| 1082607 | 9.67E-05 | 0.0009 | -7.19 | *Corynebacteriaceae; g__Corynebacterium; s__* |
| 4307230 | 0.0004 | 0.0031 | -8.45 | *Dethiosulfovibrionaceae; g__TG5; s__* |
| 4430826 | 6.23E-05 | 0.0007 | -10.22 | *Leptotrichiaceae; g__Leptotrichia; s__* |
| 4424239 | 2.15E-05 | 0.0002 | -11.87 | *Streptococcaceae; g__Streptococcus; s__* |
| 3384047 | 4.67E-06 | 8.11E-05 | -14.09 | *Streptococcaceae; g__Streptococcus; s__* |
| 4306048 | 3.05E-09 | 1.34E-07 | -16.31 | *Streptococcaceae; g__Streptococcus; s__* |
| 4340162 | 0.0002 | 0.0015 | -16.67 | *[Paraprevotellaceae]; g__[Prevotella]; s__* |
| 4455767 | 0.0009 | 0.0065 | -17.67 | *Streptococcaceae; g__Streptococcus; s__* |
| 4318672 | 0.0002 | 0.0016 | -21.13 | *Neisseriaceae; g__Neisseria; s__* |
| 4326219 | 3.70E-06 | 6.96E-05 | -21.74 | *Campylobacteraceae; g__Campylobacter; s__* |
| 3801267 | 6.68E-05 | 0.0007 | -30.02 | *Veillonellaceae; g__Veillonella; s__parvula* |
| 4294457 | 3.91E-06 | 7.10E-05 | -39.96 | *Micrococcaceae; g__Rothia; s__mucilaginosa* |
| 4307391 | 2.23E-07 | 6.17E-06 | -45.49 | *Prevotellaceae; g__Prevotella; s__melaninogenica* |
| 2613485 | 5.54E-06 | 9.05E-05 | -53.83 | *Porphyromonadaceae; g__Porphyromonas; s__* |
| 4387092 | 0.0003 | 0.0022 | -54.78 | *Fusobacteriaceae; g__Fusobacterium; s__* |
| 4319899 | 3.71E-06 | 6.96E-05 | -67.30 | *Fusobacteriaceae; g__Fusobacterium; s__* |
| 4405869 | 1.69E-09 | 9.88E-08 | -85.50 | *Fusobacteriaceae; g__Fusobacterium; s__* |
| 4446902 | 0.0006 | 0.0047 | -112.66 | *Gemellaceae; g__; s__* |
| 271159 | 1.28E-07 | 3.74E-06 | -113.61 | *Lactobacillales* |
| 12574 | 0.0003 | 0.0025 | -137.64 | *Actinomycetaceae; g__Actinomyces; s__* |
| 4453501 | 1.93E-05 | 0.0002 | -150.33 | *Veillonellaceae; g__Veillonella; s__dispar* |
| 630141 | 0.0007 | 0.0051 | -157.89 | *Staphylococcaceae; g__Staphylococcus; s__* |
| 4396235 | 1.06E-05 | 0.0001 | -483.39 | *Neisseriaceae; g__Neisseria; s__subflava* |
| 4309301 | 2.11E-06 | 4.34E-05 | -786.49 | *Streptococcaceae; g__Streptococcus; s__* |

**Table S4.**

| **KEGG Pathway; CRS, Asthma-CRS, CF-CRS, non-CRS** | **Test-Statistic** | **P** | **FDR_P** |
| --- | --- | --- | --- |
| Other glycan degradation | 16.269 | 0.001 | 0.103 |
| Various types of N-glycan biosynthesis | 15.725 | 0.001 | 0.103 |
| D-Glutamine and D-glutamate metabolism | 15.280 | 0.002 | 0.103 |
| Betalain biosynthesis | 14.046 | 0.003 | 0.103 |
| Vasopressin-regulated water reabsorption | 13.874 | 0.003 | 0.103 |
| Insulin signaling pathway | 13.874 | 0.003 | 0.103 |
| Lysosome | 13.690 | 0.003 | 0.103 |
| Melanogenesis | 13.460 | 0.004 | 0.103 |
| Glycosphingolipid biosynthesis - lacto and neolacto series | 13.355 | 0.004 | 0.103 |
| Carbohydrate digestion and absorption | 12.703 | 0.005 | 0.103 |
| Sphingolipid metabolism | 12.694 | 0.005 | 0.103 |
| Glycosphingolipid biosynthesis - globo series | 12.498 | 0.006 | 0.103 |
| Arachidonic acid metabolism | 12.454 | 0.006 | 0.103 |
| Glycosphingolipid biosynthesis - ganglio series | 12.385 | 0.006 | 0.103 |
| Nucleotide metabolism | 12.292 | 0.006 | 0.103 |
| Xylene degradation | 12.191 | 0.007 | 0.103 |
| Cell cycle - Caulobacter | 12.177 | 0.007 | 0.103 |
| Electron transfer carriers | 12.067 | 0.007 | 0.103 |
| Type I diabetes mellitus | 12.064 | 0.007 | 0.103 |
| Glycosaminoglycan degradation | 11.976 | 0.007 | 0.103 |
| Nucleotide excision repair | 11.947 | 0.008 | 0.103 |

**Table S5A.**

| **KEGG Pathway; DS I v. non-CRS** | **Test-Statistic** | **p value** | **q value** | **Fold Difference** |
| --- | --- | --- | --- | --- |
| Biosynthesis of ansamycins | 0.902 | <0.0001 | <0.0001 | 177.259 |
| Vibrio cholerae infection | 7.115 | <0.0001 | <0.0001 | -48.559 |
| Biosynthesis of type II polyketide products | 8.145 | <0.0001 | <0.0001 | -140.794 |
| DNA repair and recombination proteins | 1.003 | 0.063 | 0.075 | -210.000 |
| Alzheimer's disease | 1.139 | <0.0001 | <0.0001 | -312.912 |
| Folate biosynthesis | 1.043 | <0.0001 | <0.0001 | -596.441 |

**Table S5B.**

| **KEGG Pathway; DII v. non-CRS** | **Test-Statistic** | **p value** | **q value** | **Fold Difference** |
| --- | --- | --- | --- | --- |
| Two-component system | 0.593 | 0.0002 | 0.002 | 26656.143 |
| Bacterial motility proteins | 0.446 | 0.045 | 0.099 | 22226.071 |
| Secretion system | 0.787 | 0.024 | 0.067 | 11887.614 |
| Other ion-coupled transporters | 0.773 | 0.002 | 0.010 | 10485.914 |
| Flagellar assembly | 0.344 | 0.014 | 0.045 | 9471.471 |
| Bacterial chemotaxis | 0.416 | 0.026 | 0.069 | 8941.343 |
| Valine, leucine and isoleucine degradation | 0.641 | 0.037 | 0.087 | 7554.614 |
| Function unknown | 0.861 | 0.035 | 0.084 | 7455.586 |
| Pores ion channels | 0.688 | 0.021 | 0.059 | 6450.543 |
| Tryptophan metabolism | 0.605 | 0.021 | 0.059 | 5977.957 |
| Arginine and proline metabolism | 0.815 | 0.034 | 0.083 | 5954.214 |
| Membrane and intracellular structural molecules | 0.753 | 0.039 | 0.091 | 5587.429 |
| Fatty acid metabolism | 0.687 | 0.043 | 0.098 | 5373.371 |
| Propanoate metabolism | 0.782 | 0.043 | 0.098 | 5179.286 |
| Porphyrin and chlorophyll metabolism | 0.797 | 0.028 | 0.071 | 4818.071 |
| Lysine degradation | 0.630 | 0.015 | 0.046 | 4521.486 |
| Limonene and pinene degradation | 0.549 | 0.010 | 0.034 | 4142.129 |
| beta-Alanine metabolism | 0.641 | 0.026 | 0.069 | 4141.643 |
| Glyoxylate and dicarboxylate metabolism | 0.763 | 0.036 | 0.085 | 3975.214 |
| Protein kinases | 0.724 | 0.035 | 0.084 | 3647.000 |
| Biosynthesis of unsaturated fatty acids | 0.569 | 0.001 | 0.008 | 3490.229 |
| Pertussis | 0.279 | 0.010 | 0.033 | 2888.500 |
| Lipopolysaccharide biosynthesis | 0.746 | <0.0001 | <0.0001 | 2815.443 |
| Nitrogen metabolism | 0.876 | 0.007 | 0.025 | 2743.957 |
| Tyrosine metabolism | 0.811 | 0.030 | 0.076 | 2458.671 |
| Sulfur relay system | 0.843 | 0.034 | 0.082 | 1658.086 |
| Other transporters | 0.793 | 0.029 | 0.073 | 1594.614 |
| Metabolism of cofactors and vitamins | 0.758 | 0.001 | 0.005 | 1533.614 |
| Atrazine degradation | 0.377 | 0.018 | 0.053 | 1266.214 |
| Amino acid metabolism | 0.761 | 0.026 | 0.069 | 1248.429 |
| Arachidonic acid metabolism | 0.564 | 0.001 | 0.005 | 1107.300 |
| Vibrio cholerae pathogenic cycle | 0.684 | 0.007 | 0.026 | 1043.471 |
| beta-Lactam resistance | 0.415 | 0.012 | 0.039 | 846.514 |
| Glycan biosynthesis and metabolism | 0.644 | <0.0001 | <0.0001 | 805.300 |
| Ethylbenzene degradation | 0.589 | 0.019 | 0.055 | 803.500 |
| Cellular antigens | 0.699 | 0.044 | 0.098 | 582.357 |
| Transcription related proteins | 0.350 | 0.015 | 0.046 | 374.429 |
| Cardiac muscle contraction | 0.796 | <0.0001 | <0.0001 | 193.586 |
| Parkinson's disease | 0.897 | <0.0001 | <0.0001 | 99.971 |
| Melanogenesis | 21.000 | 0.020 | 0.058 | -1.429 |
| Betalain biosynthesis | 23.800 | 0.014 | 0.045 | -1.629 |
| Cytochrome P450 | 29.400 | 0.004 | 0.017 | -6.086 |
| Endocytosis | 97.300 | 0.002 | 0.010 | -13.757 |
| GnRH signaling pathway | 67.200 | 0.0002 | 0.002 | -14.186 |
| Fc gamma R-mediated phagocytosis | 112.000 | 0.001 | 0.004 | -15.857 |
| Bile secretion | 116.900 | <0.0001 | <0.0001 | -16.557 |
| Various types of N-glycan biosynthesis | 35.933 | <0.0001 | 0.001 | -22.457 |
| Caffeine metabolism | 25.900 | 0.0004 | 0.003 | -24.900 |
| Germination | 7.382 | 0.022 | 0.062 | -30.086 |
| Steroid biosynthesis | 11.310 | 0.0003 | 0.002 | -37.557 |
| Flavone and flavonol biosynthesis | 30.200 | <0.0001 | <0.0001 | -102.200 |
| Basal transcription factors | 34.300 | <0.0001 | <0.0001 | -128.443 |
| Type II diabetes mellitus | 1.201 | 0.003 | 0.014 | -224.514 |
| Apoptosis | 12.751 | 0.001 | 0.004 | -389.457 |
| Zeatin biosynthesis | 1.504 | 0.006 | 0.021 | -462.757 |
| Biosynthesis of ansamycins | 1.466 | 0.026 | 0.069 | -520.443 |
| Type I diabetes mellitus | 1.534 | 0.007 | 0.025 | -524.029 |
| Primary immunodeficiency | 1.559 | <0.0001 | <0.0001 | -642.857 |
| Phosphatidylinositol signaling system | 1.281 | 0.005 | 0.018 | -725.829 |
| Vitamin B6 metabolism | 1.184 | <0.0001 | <0.0001 | -807.071 |
| Xylene degradation | 7.310 | 0.0004 | 0.003 | -1006.829 |
| D-Glutamine and D-glutamate metabolism | 1.332 | 0.011 | 0.037 | -1077.986 |
| Dioxin degradation | 2.505 | 0.0003 | 0.002 | -1126.743 |
| Sphingolipid metabolism | 2.374 | 0.033 | 0.081 | -1182.329 |
| Tuberculosis | 1.463 | <0.0001 | <0.0001 | -1267.500 |
| Cytoskeleton proteins | 1.360 | 0.003 | 0.014 | -1451.743 |
| Nicotinate and nicotinamide metabolism | 1.158 | 0.025 | 0.068 | -1557.800 |
| RNA polymerase | 1.488 | 0.003 | 0.014 | -1699.557 |
| Drug metabolism - other enzymes | 1.404 | 0.001 | 0.008 | -2248.686 |
| Prenyltransferases | 1.342 | 0.018 | 0.054 | -2476.229 |
| Streptomycin biosynthesis | 1.512 | 0.001 | 0.004 | -2494.243 |
| Other glycan degradation | 3.993 | 0.014 | 0.045 | -2507.229 |
| Alanine, aspartate and glutamate metabolism | 1.120 | 0.0003 | 0.002 | -2578.200 |
| Methane metabolism | 1.122 | 0.029 | 0.073 | -2763.314 |
| Photosynthesis proteins | 1.414 | 0.008 | 0.027 | -2939.214 |
| Pantothenate and CoA biosynthesis | 1.225 | 0.006 | 0.021 | -2978.400 |
| Photosynthesis | 1.450 | 0.002 | 0.011 | -3005.443 |
| Pentose phosphate pathway | 1.193 | 0.002 | 0.010 | -3140.686 |
| Lysine biosynthesis | 1.240 | <0.0001 | <0.0001 | -3335.657 |
| Base excision repair | 1.338 | <0.0001 | 0.001 | -3395.157 |
| One carbon pool by folate | 1.263 | 0.002 | 0.011 | -3464.086 |
| Nucleotide excision repair | 1.503 | <0.0001 | <0.0001 | -3531.014 |
| Translation factors | 1.329 | 0.002 | 0.010 | -3619.586 |
| Terpenoid backbone biosynthesis | 1.296 | 0.008 | 0.027 | -3710.171 |
| Cell cycle - Caulobacter | 1.381 | 0.0003 | 0.002 | -3786.614 |
| Phenylalanine, tyrosine and tryptophan biosynthesis | 1.218 | 0.0004 | 0.003 | -3823.929 |
| Translation proteins | 1.182 | 0.028 | 0.071 | -3886.000 |
| Valine, leucine and isoleucine biosynthesis | 1.259 | 0.014 | 0.045 | -4277.486 |
| Fructose and mannose metabolism | 1.330 | 0.002 | 0.011 | -4558.657 |
| Starch and sucrose metabolism | 1.385 | 0.001 | 0.007 | -5010.886 |
| DNA replication | 1.379 | 0.004 | 0.017 | -5133.343 |
| Mismatch repair | 1.326 | 0.012 | 0.039 | -5273.243 |
| Peptidoglycan biosynthesis | 1.303 | 0.015 | 0.046 | -5490.286 |
| Protein export | 1.434 | 0.003 | 0.014 | -5519.986 |
| Peptidases | 1.143 | 0.043 | 0.098 | -5621.571 |
| Galactose metabolism | 1.801 | 0.003 | 0.014 | -6532.129 |
| Homologous recombination | 1.358 | 0.005 | 0.019 | -6838.129 |
| Ribosome Biogenesis | 1.197 | 0.017 | 0.050 | -6901.043 |
| Amino acid related enzymes | 1.224 | <0.0001 | 0.001 | -7130.800 |
| DNA replication proteins | 1.336 | 0.004 | 0.017 | -7874.714 |
| Amino sugar and nucleotide sugar metabolism | 1.297 | 0.003 | 0.014 | -7916.729 |
| Aminoacyl-tRNA biosynthesis | 1.463 | 0.0003 | 0.002 | -10834.329 |
| Pyrimidine metabolism | 1.415 | 0.001 | 0.008 | -14619.400 |
| Purine metabolism | 1.285 | 0.001 | 0.003 | -14627.371 |
| DNA repair and recombination proteins | 1.289 | 0.001 | 0.008 | -18016.643 |
| Ribosome | 1.463 | 0.002 | 0.011 | -21194.914 |

**Table S5C.**

| **KEGG Pathway; DS IIIa v. non-CRS** | **Test-Statistic** | **p value** | **q value** | **Fold Difference** |
| --- | --- | --- | --- | --- |
| Other ion-coupled transporters | 0.748 | 0.001 | 0.006 | 12036.478 |
| ABC transporters | 0.932 | 0.019 | 0.074 | 6601.422 |
| Histidine metabolism | 0.701 | <0.0001 | <0.0001 | 5058.233 |
| Tryptophan metabolism | 0.663 | 0.022 | 0.082 | 4649.822 |
| Porphyrin and chlorophyll metabolism | 0.804 | 0.009 | 0.043 | 4609.444 |
| Butanoate metabolism | 0.835 | 0.010 | 0.045 | 4019.600 |
| Limonene and pinene degradation | 0.586 | 0.020 | 0.075 | 3565.756 |
| Ubiquinone and other terpenoid-quinone biosynthesis | 0.703 | <0.0001 | <0.0001 | 3426.178 |
| Glycine, serine and threonine metabolism | 0.887 | <0.0001 | <0.0001 | 2979.222 |
| Alanine, aspartate and glutamate metabolism | 0.892 | 0.003 | 0.017 | 2926.189 |
| Phenylalanine metabolism | 0.642 | 0.017 | 0.070 | 2887.167 |
| Sulfur relay system | 0.784 | <0.0001 | 0.0001 | 2446.800 |
| Ascorbate and aldarate metabolism | 0.598 | <0.0001 | 0.001 | 2157.789 |
| Peroxisome | 0.750 | <0.0001 | 0.001 | 2018.667 |
| PPAR signaling pathway | 0.694 | 0.003 | 0.018 | 1832.922 |
| Sulfur metabolism | 0.819 | 0.0001 | 0.001 | 1813.056 |
| Riboflavin metabolism | 0.832 | 0.003 | 0.015 | 1466.822 |
| Tyrosine metabolism | 0.878 | 0.019 | 0.074 | 1456.544 |
| Adipocytokine signaling pathway | 0.694 | 0.011 | 0.051 | 1081.178 |
| Toluene degradation | 0.813 | 0.0003 | 0.003 | 945.800 |
| Ethylbenzene degradation | 0.551 | <0.0001 | 0.0003 | 941.167 |
| Proximal tubule bicarbonate reclamation | 0.474 | <0.0001 | 0.001 | 809.067 |
| Ubiquitin system | 0.526 | 0.001 | 0.005 | 723.300 |
| Carotenoid biosynthesis | 0.497 | 0.019 | 0.075 | 648.922 |
| Novobiocin biosynthesis | 0.833 | 0.002 | 0.011 | 576.156 |
| Phosphonate and phosphinate metabolism | 0.754 | 0.005 | 0.027 | 567.211 |
| Amyotrophic lateral sclerosis (ALS) | 0.606 | 0.006 | 0.028 | 532.844 |
| Basal transcription factors | 0.201 | 0.0002 | 0.002 | 526.367 |
| Chagas disease (American trypanosomiasis) | 0.452 | 0.0001 | 0.002 | 441.900 |
| Meiosis - yeast | 0.528 | 0.003 | 0.018 | 435.300 |
| African trypanosomiasis | 0.490 | 0.002 | 0.011 | 427.744 |
| Proteasome | 0.705 | 0.020 | 0.075 | 413.822 |
| alpha-Linolenic acid metabolism | 0.520 | 0.014 | 0.059 | 402.567 |
| Lipoic acid metabolism | 0.854 | 0.025 | 0.092 | 333.400 |
| Renal cell carcinoma | 0.705 | <0.0001 | 0.0004 | 313.722 |
| RIG-I-like receptor signaling pathway | 0.410 | 0.015 | 0.062 | 269.478 |
| N-Glycan biosynthesis | 0.695 | 0.029 | 0.099 | 254.311 |
| Steroid biosynthesis | 0.351 | 0.029 | 0.098 | 76.244 |
| Betalain biosynthesis | 0.097 | 0.0005 | 0.004 | 15.744 |
| Melanogenesis | 0.099 | 0.001 | 0.006 | 13.611 |
| Glycosphingolipid biosynthesis - lacto and neolacto series | 0.087 | <0.0001 | <0.0001 | 3.700 |
| Fatty acid elongation in mitochondria | 9.477 | <0.0001 | 0.000 | -0.844 |
| Various types of N-glycan biosynthesis | 25.988 | 0.001 | 0.009 | -22.211 |
| Bacterial invasion of epithelial cells | 7.217 | <0.0001 | <0.0001 | -73.911 |
| Flavone and flavonol biosynthesis | 14.199 | <0.0001 | 0.001 | -98.256 |
| Systemic lupus erythematosus | 6.166 | 0.001 | 0.006 | -126.844 |
| Biosynthesis of type II polyketide products | 6.449 | 0.019 | 0.075 | -135.611 |
| Type II diabetes mellitus | 1.122 | 0.024 | 0.089 | -145.689 |
| Protein digestion and absorption | 3.657 | 0.002 | 0.011 | -214.911 |
| NOD-like receptor signaling pathway | 1.797 | 0.0003 | 0.002 | -222.222 |
| Zeatin biosynthesis | 1.211 | 0.026 | 0.094 | -240.233 |
| Apoptosis | 2.432 | 0.006 | 0.030 | -248.822 |
| Ion channels | 1.626 | 0.005 | 0.025 | -565.578 |
| Glycosphingolipid biosynthesis - ganglio series | 4.224 | <0.0001 | 0.0002 | -598.922 |
| Cyanoamino acid metabolism | 1.122 | 0.027 | 0.096 | -616.089 |
| Xylene degradation | 2.140 | 0.0003 | 0.003 | -621.400 |
| Epithelial cell signaling in Helicobacter pylori infection | 1.709 | 0.008 | 0.038 | -621.722 |
| MAPK signaling pathway - yeast | 2.389 | <0.0001 | <0.0001 | -644.300 |
| Dioxin degradation | 1.814 | 0.002 | 0.015 | -841.822 |
| Glycosaminoglycan degradation | 4.826 | <0.0001 | <0.0001 | -903.878 |
| Glycosphingolipid biosynthesis - globo series | 3.587 | 0.0003 | 0.003 | -926.589 |
| Lysosome | 3.905 | <0.0001 | 0.0004 | -1045.989 |
| Bacterial toxins | 1.448 | <0.0001 | 0.001 | -1103.411 |
| Sphingolipid metabolism | 2.516 | <0.0001 | 0.0003 | -1230.789 |
| Restriction enzyme | 1.493 | 0.014 | 0.060 | -1321.111 |
| Cell motility and secretion | 1.456 | 0.002 | 0.012 | -1656.989 |
| Lipid biosynthesis proteins | 1.103 | 0.009 | 0.044 | -1751.544 |
| Tetracycline biosynthesis | 1.739 | 0.0002 | 0.002 | -1761.867 |
| RNA transport | 2.345 | <0.0001 | <0.0001 | -1849.889 |
| Cell cycle - Caulobacter | 1.156 | 0.005 | 0.028 | -1853.733 |
| Glycerophospholipid metabolism | 1.145 | 0.0001 | 0.001 | -1904.411 |
| Other glycan degradation | 3.850 | 0.0001 | 0.001 | -2476.022 |
| Membrane and intracellular structural molecules | 1.275 | 0.028 | 0.098 | -3668.722 |
| Peptidoglycan biosynthesis | 1.184 | 0.018 | 0.074 | -3675.000 |
| Lipopolysaccharide biosynthesis | 1.873 | 0.006 | 0.030 | -3856.311 |
| Chaperones and folding catalysts | 1.176 | <0.0001 | 0.0002 | -4174.611 |
| Translation proteins | 1.204 | 0.0002 | 0.002 | -4278.556 |
| Fatty acid biosynthesis | 1.444 | <0.0001 | <0.0001 | -4418.856 |
| DNA replication proteins | 1.188 | 0.010 | 0.044 | -4956.611 |
| Lipopolysaccharide biosynthesis proteins | 1.780 | 0.004 | 0.020 | -5051.678 |
| Chromosome | 1.192 | <0.0001 | 0.0004 | -6410.111 |
| Ribosome Biogenesis | 1.204 | 0.0004 | 0.003 | -7118.900 |

**Table S5D.**

| **KEGG Pathway; DSIIIb vs. non-CRS** | **Test-Statistic** | **p value** | **q value** | **Fold Difference** |
| --- | --- | --- | --- | --- |
| Two-component system | 0.678 | 0.0002 | 0.002 | 18455.395 |
| Bacterial motility proteins | 0.562 | 0.029 | 0.073 | 13903.395 |
| ABC transporters | 0.869 | 0.0002 | 0.002 | 13623.253 |
| Valine, leucine and isoleucine degradation | 0.668 | 0.008 | 0.026 | 6719.663 |
| Flagellar assembly | 0.445 | 0.013 | 0.038 | 6217.453 |
| Glyoxylate and dicarboxylate metabolism | 0.676 | 0.0001 | 0.002 | 6107.658 |
| Propanoate metabolism | 0.754 | 0.0003 | 0.002 | 6059.579 |
| Butanoate metabolism | 0.781 | 0.0002 | 0.002 | 5704.653 |
| Bacterial chemotaxis | 0.535 | 0.017 | 0.047 | 5548.095 |
| Fatty acid metabolism | 0.684 | 0.008 | 0.026 | 5445.274 |
| Benzoate degradation | 0.640 | 0.007 | 0.023 | 5124.937 |
| Transcription factors | 0.883 | 0.008 | 0.026 | 5122.316 |
| Tryptophan metabolism | 0.649 | 0.005 | 0.020 | 4940.705 |
| Lysine degradation | 0.652 | 0.005 | 0.018 | 4114.253 |
| Limonene and pinene degradation | 0.555 | 0.002 | 0.007 | 4047.095 |
| Aminobenzoate degradation | 0.641 | 0.001 | 0.007 | 3922.553 |
| Pyruvate metabolism | 0.889 | 0.032 | 0.078 | 3681.621 |
| beta-Alanine metabolism | 0.672 | 0.013 | 0.038 | 3603.605 |
| Protein kinases | 0.751 | 0.001 | 0.005 | 3171.447 |
| Pentose and glucuronate interconversions | 0.730 | 0.001 | 0.006 | 2711.979 |
| Phenylalanine metabolism | 0.677 | 0.037 | 0.086 | 2466.132 |
| Chloroalkane and chloroalkene degradation | 0.678 | 0.001 | 0.007 | 2329.974 |
| Drug metabolism - cytochrome P450 | 0.589 | 0.003 | 0.011 | 2202.189 |
| Metabolism of xenobiotics by cytochrome P450 | 0.591 | 0.002 | 0.010 | 2126.374 |
| Naphthalene degradation | 0.709 | 0.001 | 0.006 | 2095.068 |
| Signal transduction mechanisms | 0.852 | <0.0001 | <0.0001 | 2079.884 |
| Ascorbate and aldarate metabolism | 0.638 | 0.0004 | 0.003 | 1819.637 |
| Nitrogen metabolism | 0.921 | 0.033 | 0.080 | 1662.837 |
| Synthesis and degradation of ketone bodies | 0.608 | 0.004 | 0.015 | 1609.400 |
| Biosynthesis of unsaturated fatty acids | 0.749 | 0.016 | 0.045 | 1546.142 |
| Chlorocyclohexane and chlorobenzene degradation | 0.401 | 0.001 | 0.006 | 1542.832 |
| Atrazine degradation | 0.340 | 0.0002 | 0.002 | 1486.789 |
| Tyrosine metabolism | 0.879 | 0.033 | 0.080 | 1446.416 |
| Sulfur metabolism | 0.854 | 0.008 | 0.026 | 1404.342 |
| Sulfur relay system | 0.872 | 0.034 | 0.080 | 1301.379 |
| Carotenoid biosynthesis | 0.343 | 0.001 | 0.003 | 1226.437 |
| Bisphenol degradation | 0.586 | 0.018 | 0.049 | 1138.747 |
| Amino acid metabolism | 0.783 | <0.0001 | 0.001 | 1100.789 |
| beta-Lactam resistance | 0.362 | <0.0001 | 0.001 | 1056.905 |
| Toluene degradation | 0.803 | 0.004 | 0.017 | 1010.695 |
| Ethylbenzene degradation | 0.572 | <0.0001 | 0.0004 | 863.500 |
| Retinol metabolism | 0.707 | 0.022 | 0.059 | 751.342 |
| Arachidonic acid metabolism | 0.676 | 0.002 | 0.007 | 687.432 |
| Nitrotoluene degradation | 0.594 | 0.038 | 0.088 | 621.916 |
| Linoleic acid metabolism | 0.626 | 0.035 | 0.083 | 484.000 |
| Primary bile acid biosynthesis | 0.333 | 0.0002 | 0.002 | 410.700 |
| Amyotrophic lateral sclerosis (ALS) | 0.701 | 0.037 | 0.086 | 349.611 |
| D-Arginine and D-ornithine metabolism | 0.258 | <0.0001 | <0.0001 | 344.616 |
| alpha-Linolenic acid metabolism | 0.582 | 0.024 | 0.062 | 313.216 |
| Secondary bile acid biosynthesis | 0.234 | 0.0002 | 0.002 | 278.211 |
| Ether lipid metabolism | 0.500 | 0.025 | 0.065 | 222.237 |
| Prion diseases | 0.494 | 0.013 | 0.038 | 142.589 |
| Amoebiasis | 0.460 | 0.009 | 0.027 | 130.258 |
| Steroid biosynthesis | 0.273 | 0.003 | 0.011 | 109.537 |
| Betalain biosynthesis | 0.061 | <0.0001 | <0.0001 | 26.089 |
| Melanogenesis | 0.099 | <0.0001 | 0.0001 | 13.605 |
| Various types of N-glycan biosynthesis | 9.975 | 0.029 | 0.073 | -20.784 |
| Systemic lupus erythematosus | 3.288 | 0.002 | 0.009 | -105.347 |
| Protein digestion and absorption | 3.790 | 0.001 | 0.004 | -217.747 |
| Type II diabetes mellitus | 1.232 | 0.0004 | 0.003 | -252.695 |
| Butirosin and neomycin biosynthesis | 1.293 | 0.008 | 0.025 | -264.747 |
| Carbohydrate digestion and absorption | 2.350 | 0.006 | 0.022 | -315.611 |
| Ribosome biogenesis in eukaryotes | 1.242 | 0.004 | 0.016 | -361.974 |
| Primary immunodeficiency | 1.258 | 0.007 | 0.023 | -367.579 |
| Type I diabetes mellitus | 1.345 | 0.001 | 0.004 | -386.100 |
| Zeatin biosynthesis | 1.435 | 0.0001 | 0.001 | -418.374 |
| Biosynthesis of vancomycin group antibiotics | 1.555 | 0.013 | 0.038 | -434.495 |
| Phosphatidylinositol signaling system | 1.191 | 0.009 | 0.028 | -531.400 |
| Glutamatergic synapse | 1.307 | 0.003 | 0.012 | -568.811 |
| Ion channels | 1.659 | 0.002 | 0.008 | -583.589 |
| Insulin signaling pathway | 1.424 | 0.029 | 0.073 | -588.405 |
| Vitamin B6 metabolism | 1.131 | 0.002 | 0.007 | -599.684 |
| Glycosphingolipid biosynthesis - ganglio series | 5.296 | <0.0001 | <0.0001 | -636.542 |
| Glycosphingolipid biosynthesis - globo series | 2.531 | 0.006 | 0.021 | -777.174 |
| Glycosaminoglycan degradation | 3.392 | 0.002 | 0.008 | -803.942 |
| D-Glutamine and D-glutamate metabolism | 1.288 | <0.0001 | 0.001 | -966.121 |
| Lysosome | 3.270 | 0.001 | 0.004 | -976.047 |
| Bacterial toxins | 1.382 | 0.001 | 0.004 | -986.721 |
| Sphingolipid metabolism | 2.050 | 0.003 | 0.013 | -1046.163 |
| RNA polymerase | 1.280 | 0.006 | 0.021 | -1133.832 |
| Restriction enzyme | 1.507 | 0.0002 | 0.002 | -1345.263 |
| Carbon fixation in photosynthetic organisms | 1.108 | 0.030 | 0.074 | -1356.037 |
| Prenyltransferases | 1.167 | 0.021 | 0.058 | -1392.537 |
| Glycosyltransferases | 1.148 | 0.016 | 0.045 | -1474.168 |
| RNA degradation | 1.138 | 0.007 | 0.024 | -1546.868 |
| Alanine, aspartate and glutamate metabolism | 1.071 | 0.025 | 0.065 | -1592.121 |
| Drug metabolism - other enzymes | 1.258 | 0.014 | 0.040 | -1600.847 |
| Transcription machinery | 1.096 | 0.029 | 0.073 | -1685.268 |
| Polyketide sugar unit biosynthesis | 1.686 | 0.001 | 0.007 | -1734.495 |
| Nicotinate and nicotinamide metabolism | 1.191 | 0.0001 | 0.001 | -1832.142 |
| Base excision repair | 1.167 | 0.003 | 0.012 | -1920.142 |
| Lysine biosynthesis | 1.146 | 0.0003 | 0.002 | -2192.932 |
| Cell cycle - Caulobacter | 1.192 | 0.001 | 0.003 | -2213.611 |
| Streptomycin biosynthesis | 1.442 | 0.0002 | 0.002 | -2255.837 |
| Other glycan degradation | 3.602 | <0.0001 | 0.0001 | -2416.168 |
| Nucleotide excision repair | 1.329 | <0.0001 | 0.0002 | -2611.642 |
| Terpenoid backbone biosynthesis | 1.201 | 0.0005 | 0.003 | -2716.153 |
| Phenylalanine, tyrosine and tryptophan biosynthesis | 1.146 | 0.001 | 0.003 | -2731.842 |
| Translation factors | 1.310 | <0.0001 | <0.0001 | -3456.800 |
| Protein export | 1.234 | 0.004 | 0.016 | -3459.805 |
| Chaperones and folding catalysts | 1.142 | 0.013 | 0.038 | -3472.658 |
| DNA replication | 1.253 | 0.002 | 0.009 | -3770.884 |
| Peptidoglycan biosynthesis | 1.191 | 0.029 | 0.073 | -3784.474 |
| One carbon pool by folate | 1.316 | <0.0001 | <0.0001 | -4001.958 |
| Chromosome | 1.115 | 0.008 | 0.025 | -4091.316 |
| Mismatch repair | 1.241 | 0.001 | 0.006 | -4157.442 |
| Translation proteins | 1.214 | 0.0001 | 0.001 | -4450.947 |
| Homologous recombination | 1.285 | 0.0004 | 0.003 | -5753.963 |
| Amino acid related enzymes | 1.177 | <0.0001 | 0.0004 | -5857.142 |
| DNA replication proteins | 1.244 | 0.001 | 0.007 | -6140.763 |
| Peptidases | 1.170 | <0.0001 | 0.0002 | -6559.579 |
| Amino sugar and nucleotide sugar metabolism | 1.259 | 0.001 | 0.006 | -7100.958 |
| Aminoacyl-tRNA biosynthesis | 1.297 | 0.0001 | 0.001 | -7845.874 |
| Ribosome Biogenesis | 1.244 | 0.0003 | 0.002 | -8238.532 |
| Purine metabolism | 1.192 | 0.0003 | 0.002 | -10597.247 |
| Pyrimidine metabolism | 1.303 | 0.0001 | 0.002 | -11601.058 |
| DNA repair and recombination proteins | 1.215 | 0.0001 | 0.002 | -14214.526 |
| Ribosome | 1.326 | 0.0002 | 0.002 | -16446.253 |
|  |  |  |  |  |

**Supplemental References.**

1 Caporaso, J. G. *et al.* QIIME allows analysis of high-throughput community sequencing data. *Nat Methods* **7**, 335-336, doi:nmeth.f.303 [pii]

10.1038/nmeth.f.303 (2010).

2 Kang, D. W. *et al.* Microbiota Transfer Therapy alters gut ecosystem and improves gastrointestinal and autism symptoms: an open-label study. *Microbiome* **5**, 10, doi:10.1186/s40168-016-0225-7 (2017).

3 Halfvarson, J. *et al.* Dynamics of the human gut microbiome in inflammatory bowel disease. *Nature microbiology* **2**, 17004, doi:10.1038/nmicrobiol.2017.4 (2017).

4 Magoč, T. & Salzberg, S. L. FLASH: fast length adjustment of short reads to improve genome assemblies. *Bioinformatics* **27**, 2957-2963, doi:10.1093/bioinformatics/btr507 (2011).

5 DeSantis, T. Z. *et al.* Greengenes, a chimera-checked 16S rRNA gene database and workbench compatible with ARB. *Appl Environ Microbiol* **72**, 5069-5072, doi:72/7/5069 [pii]

10.1128/AEM.03006-05 (2006).

6 Edgar, R. C. Search and clustering orders of magnitude faster than BLAST. *Bioinformatics* **26**, 2460-2461, doi:btq461 [pii]

10.1093/bioinformatics/btq461 (2010).

7 Caporaso, J. G. *et al.* PyNAST: a flexible tool for aligning sequences to a template alignment. *Bioinformatics* **26**, 266-267, doi:btp636 [pii]

10.1093/bioinformatics/btp636 (2010).

8 Salter, S. J. *et al.* Reagent and laboratory contamination can critically impact sequence-based microbiome analyses. *BMC biology* **12**, 87, doi:10.1186/s12915-014-0087-z (2014).

9 Price, M. N., Dehal, P. S. & Arkin, A. P. FastTree: computing large minimum evolution trees with profiles instead of a distance matrix. *Mol Biol Evol* **26**, 1641-1650, doi:msp077 [pii]

10.1093/molbev/msp077 (2009).

10 Langille, M. G. *et al.* Predictive functional profiling of microbial communities using 16S rRNA marker gene sequences. *Nat Biotechnol*, doi:nbt.2676 [pii]

10.1038/nbt.2676 (2013).

11 Lane, D. *16S/23S rRNA sequencing.*, pp 115–175. (John Wiley & Sons. , 1991).

12 Roediger, F. C. *et al.* Nucleic acid extraction efficiency and bacterial recovery from maxillary sinus mucosal samples obtained by brushing or biopsy. *Am J Rhinol Allergy* **24**, 263-265, doi:10.2500/ajra.2010.24.3472 (2010).

13 Einen, J., Thorseth, I. H. & Ovreas, L. Enumeration of Archaea and Bacteria in seafloor basalt using real-time quantitative PCR and fluorescence microscopy. *FEMS Microbiol Lett* **282**, 182-187, doi:10.1111/j.1574-6968.2008.01119.x (2008).

14 Livak, K. J. & Schmittgen, T. D. Analysis of relative gene expression data using real-time quantitative PCR and the 2(-Delta Delta C(T)) Method. *Methods* **25**, 402-408, doi:10.1006/meth.2001.1262

S1046-2023(01)91262-9 [pii] (2001).
