# Supplementary figures and images for: Compositionally and functionally distinct sinus microbiota in chronic rhinosinusitis patients have immunological and clinically divergent consequences
Source: Microbiome. 2017 May 12;5:53. doi: 10.1186/s40168-017-0266-6 (PMC5427582; doi:10.1186/s40168-017-0266-6)

A.

Lund McKay Score

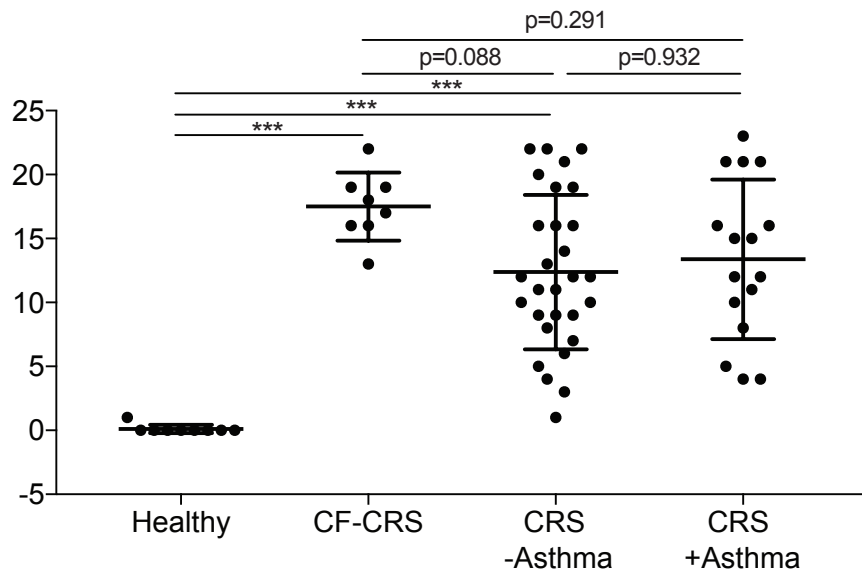

B.

Age

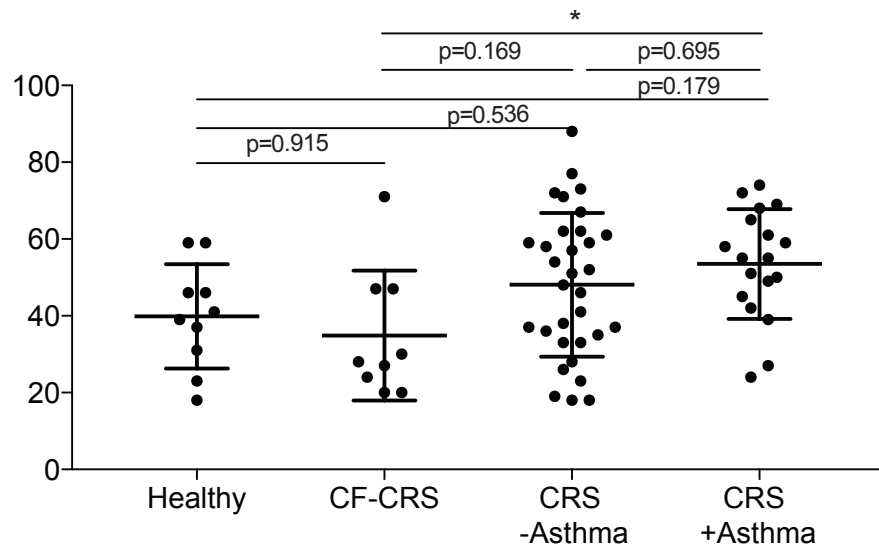

\*\*\*p&lt;0.001, \*\*p&lt;0.01, \*p&lt;0.05

Supplement: Supplementary file 6 — Supplemental Information. Figure S1A. Lund-MacKay scores associated with disease state. No differences were observed between CRS, CRS+A or CRS+CF patients; B. CRS-CF patients are significantly younger than CRS+A patients (ANOVA, Tukey’s p = 0.041); however, no differences in age were observed for pairwise comparisons between the other groups (p > 0.05, Tukey’s post hoc test). Figure S2A. PCoA of an unweighted UniFrac distance matrix colored by DSI-IIIb and healthy (PERMANOVA p = 0.001, 18.2% variation explained); B. PCoA of weighted UniFrac distance matrix after dominant sequence reads associated with the dominant family in each sample were removed demonstrating that DS still significantly explains variation in community composition despite removal of the dominant taxon from each sample (PERMANOVA p = 0.001, 17.6% variation explained). Figure S3A. Laplace model fit demonstrates three distinct Dirichlet multinomial mixtures groups. B. Hierarchical cluster analysis using a weighted-UniFrac distance matrix showing that microbiomes enriched in Corynebacteriaceae forms a distinct cluster (au p = 100). Heatmap shows relative abundance of the bacterial general that comprise >90% of the total sequence reads. C. Reciprocal relationship between Corynebacteriaceae and Staphylococcaceae. Figure S4. PICRUSt-predicted functional variation across microbial Dirichlet states shows significant functional differences A. PCoA of Canberra distance matrix; PERMANOVA p = 0.001, 21.7% of variation explained) B. PCoA of Bray-Curtis distance matrix; PERMANOVA p = 0.001, 22.0% of variation explained). Figure S5. Expression levels of all host immune genes measured by QPCR (*indicates Kruskal-Wallis p < 0.05, q < 0.15; **indicates Kruskal-Wallis p < 0.05, q < 0.05; DS vs. nonCRS). Table S1–S5. (ZIP 1671 kb) [file 40168_2017_266_MOESM6_ESM.zip › FigS1_040617.pdf]

A.

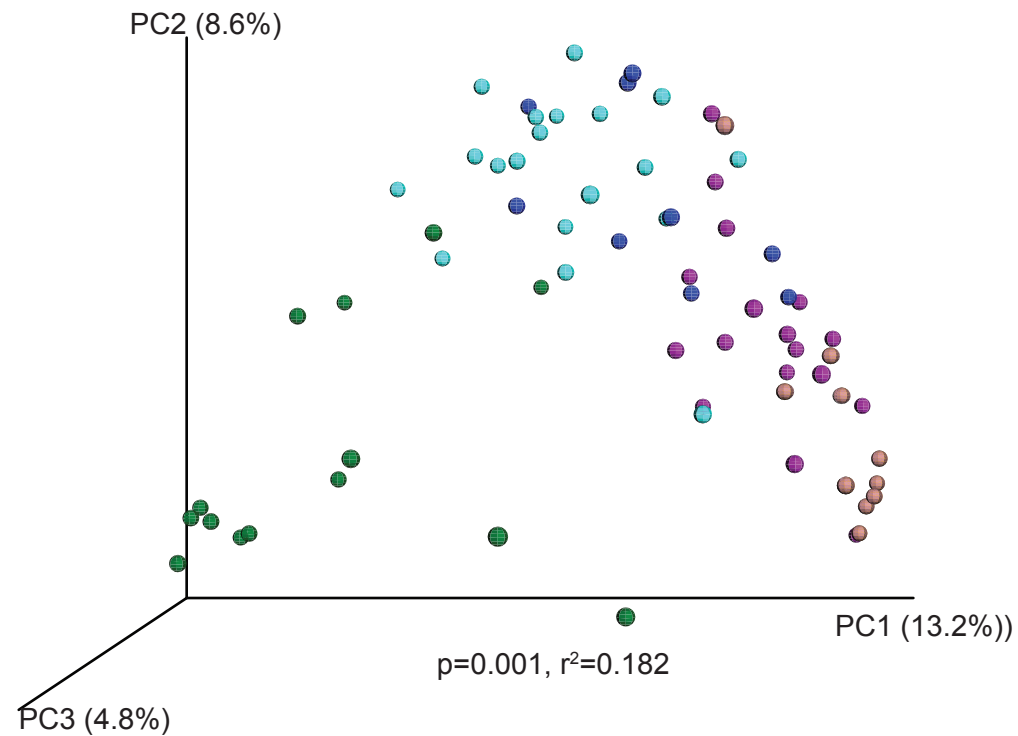

B.

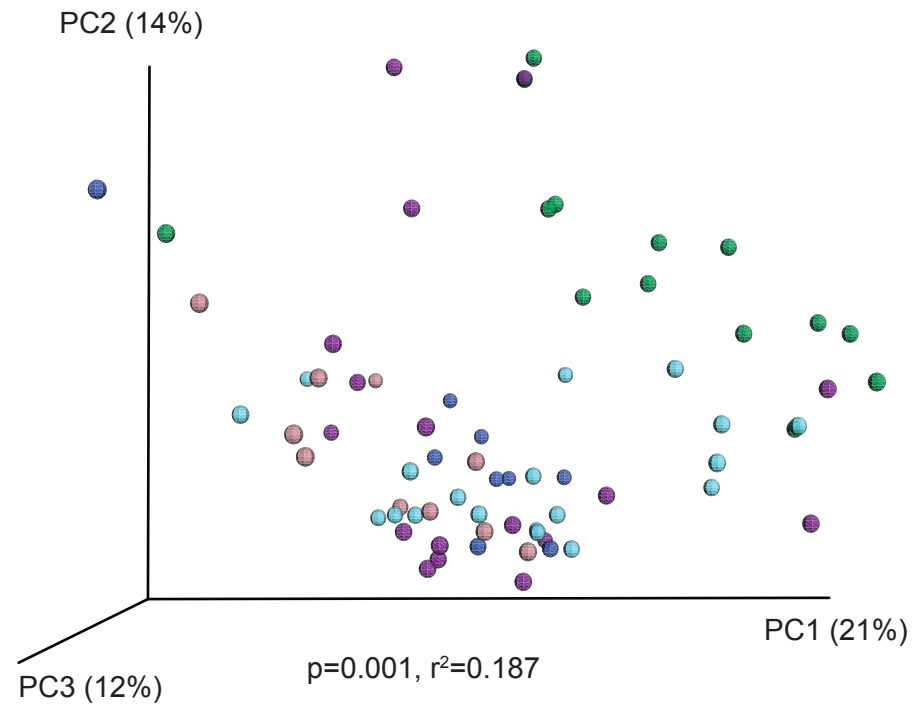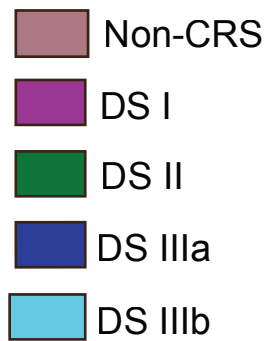

Supplement: Supplementary file 6 — Supplemental Information. Figure S1A. Lund-MacKay scores associated with disease state. No differences were observed between CRS, CRS+A or CRS+CF patients; B. CRS-CF patients are significantly younger than CRS+A patients (ANOVA, Tukey’s p = 0.041); however, no differences in age were observed for pairwise comparisons between the other groups (p > 0.05, Tukey’s post hoc test). Figure S2A. PCoA of an unweighted UniFrac distance matrix colored by DSI-IIIb and healthy (PERMANOVA p = 0.001, 18.2% variation explained); B. PCoA of weighted UniFrac distance matrix after dominant sequence reads associated with the dominant family in each sample were removed demonstrating that DS still significantly explains variation in community composition despite removal of the dominant taxon from each sample (PERMANOVA p = 0.001, 17.6% variation explained). Figure S3A. Laplace model fit demonstrates three distinct Dirichlet multinomial mixtures groups. B. Hierarchical cluster analysis using a weighted-UniFrac distance matrix showing that microbiomes enriched in Corynebacteriaceae forms a distinct cluster (au p = 100). Heatmap shows relative abundance of the bacterial general that comprise >90% of the total sequence reads. C. Reciprocal relationship between Corynebacteriaceae and Staphylococcaceae. Figure S4. PICRUSt-predicted functional variation across microbial Dirichlet states shows significant functional differences A. PCoA of Canberra distance matrix; PERMANOVA p = 0.001, 21.7% of variation explained) B. PCoA of Bray-Curtis distance matrix; PERMANOVA p = 0.001, 22.0% of variation explained). Figure S5. Expression levels of all host immune genes measured by QPCR (*indicates Kruskal-Wallis p < 0.05, q < 0.15; **indicates Kruskal-Wallis p < 0.05, q < 0.05; DS vs. nonCRS). Table S1–S5. (ZIP 1671 kb) [file 40168_2017_266_MOESM6_ESM.zip › FigS2_revised_040617.pdf]

A

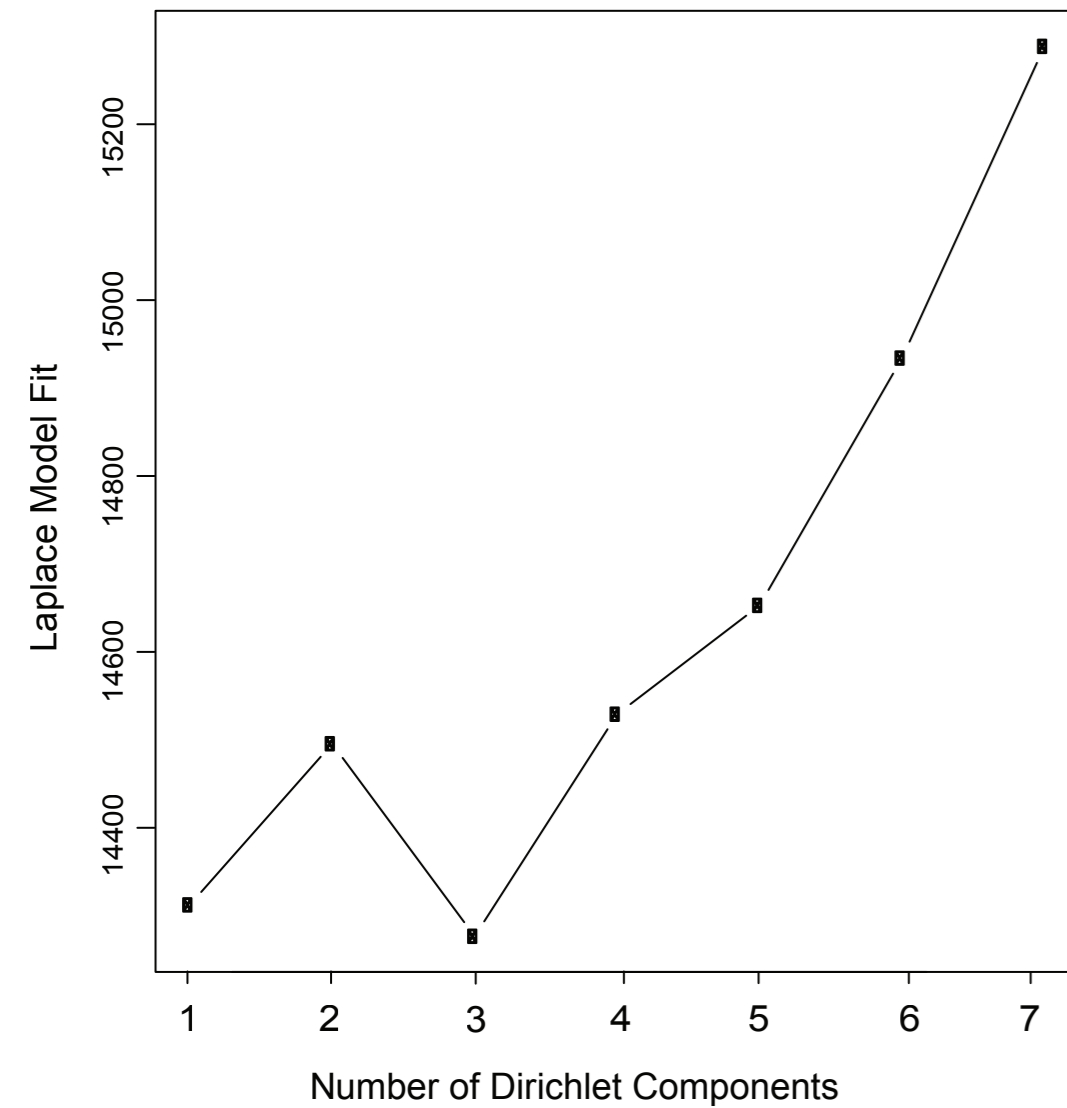

B

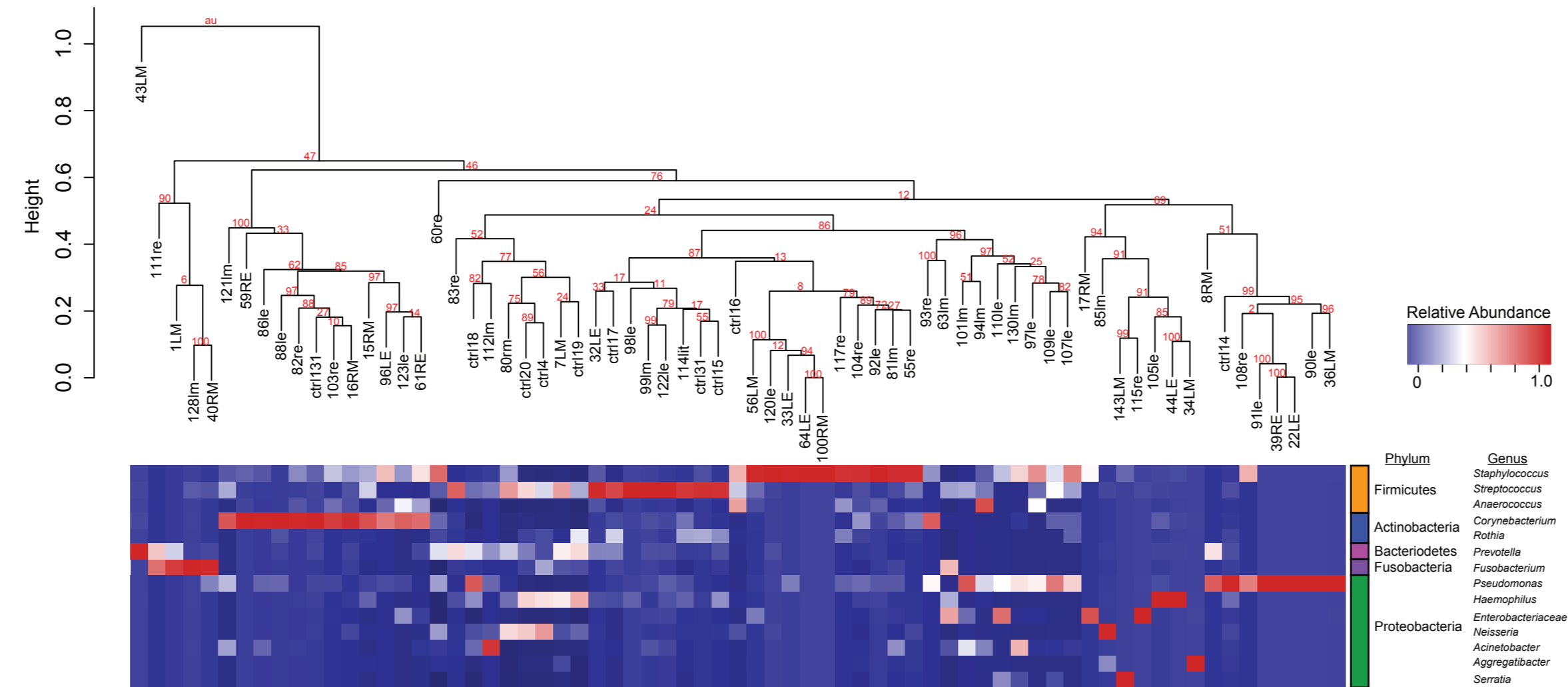

C

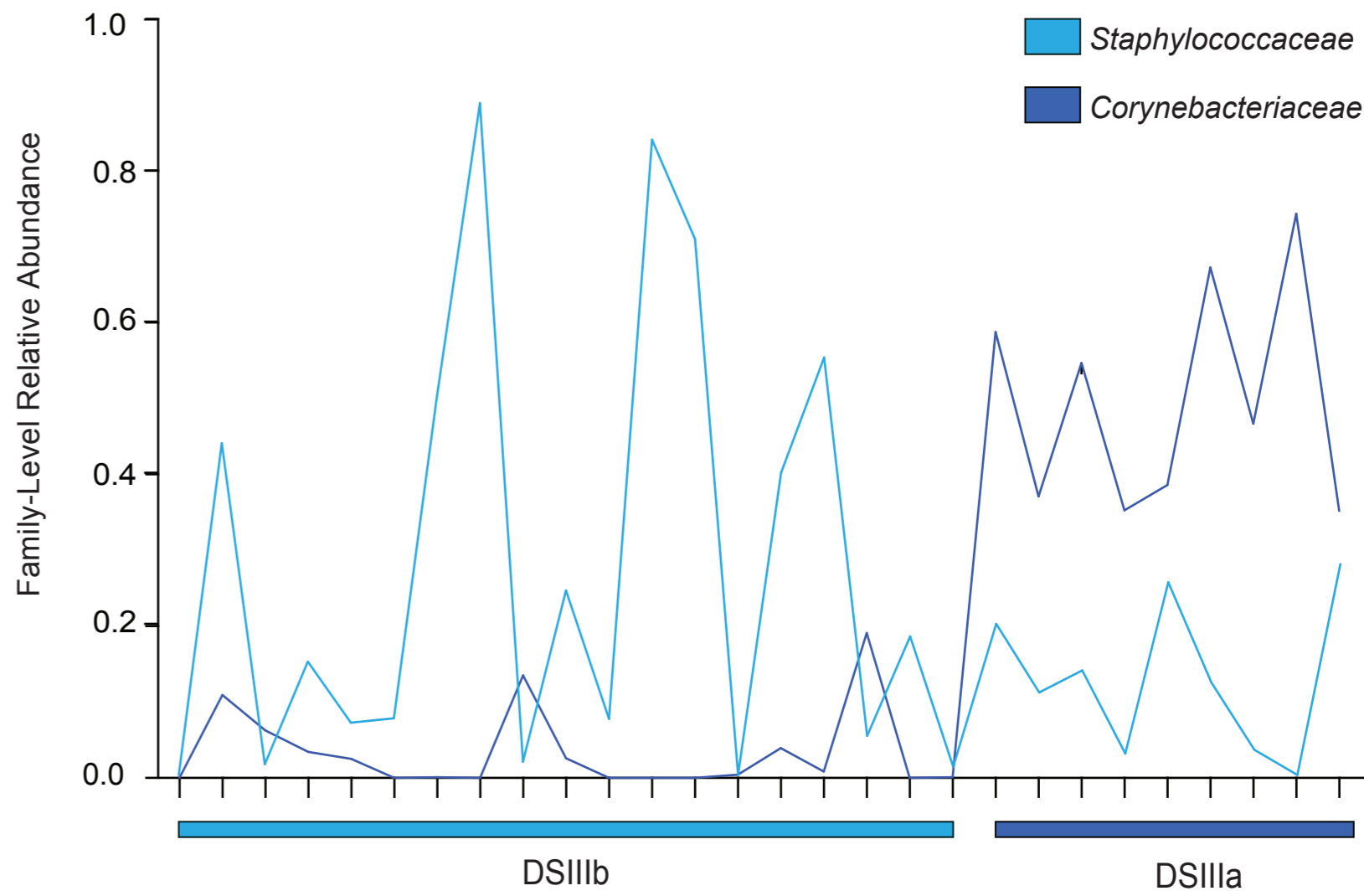

Supplement: Supplementary file 6 — Supplemental Information. Figure S1A. Lund-MacKay scores associated with disease state. No differences were observed between CRS, CRS+A or CRS+CF patients; B. CRS-CF patients are significantly younger than CRS+A patients (ANOVA, Tukey’s p = 0.041); however, no differences in age were observed for pairwise comparisons between the other groups (p > 0.05, Tukey’s post hoc test). Figure S2A. PCoA of an unweighted UniFrac distance matrix colored by DSI-IIIb and healthy (PERMANOVA p = 0.001, 18.2% variation explained); B. PCoA of weighted UniFrac distance matrix after dominant sequence reads associated with the dominant family in each sample were removed demonstrating that DS still significantly explains variation in community composition despite removal of the dominant taxon from each sample (PERMANOVA p = 0.001, 17.6% variation explained). Figure S3A. Laplace model fit demonstrates three distinct Dirichlet multinomial mixtures groups. B. Hierarchical cluster analysis using a weighted-UniFrac distance matrix showing that microbiomes enriched in Corynebacteriaceae forms a distinct cluster (au p = 100). Heatmap shows relative abundance of the bacterial general that comprise >90% of the total sequence reads. C. Reciprocal relationship between Corynebacteriaceae and Staphylococcaceae. Figure S4. PICRUSt-predicted functional variation across microbial Dirichlet states shows significant functional differences A. PCoA of Canberra distance matrix; PERMANOVA p = 0.001, 21.7% of variation explained) B. PCoA of Bray-Curtis distance matrix; PERMANOVA p = 0.001, 22.0% of variation explained). Figure S5. Expression levels of all host immune genes measured by QPCR (*indicates Kruskal-Wallis p < 0.05, q < 0.15; **indicates Kruskal-Wallis p < 0.05, q < 0.05; DS vs. nonCRS). Table S1–S5. (ZIP 1671 kb) [file 40168_2017_266_MOESM6_ESM.zip › FigS3_040617.pdf]

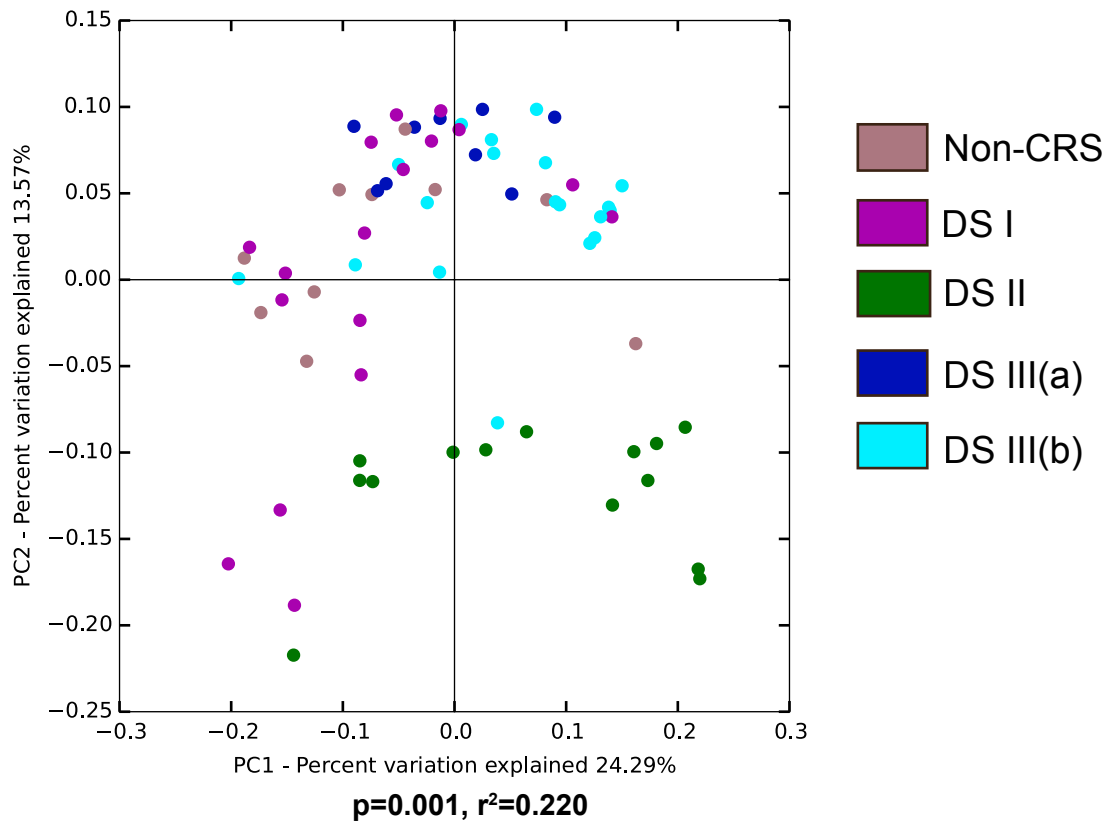

Supplement: Supplementary file 6 — Supplemental Information. Figure S1A. Lund-MacKay scores associated with disease state. No differences were observed between CRS, CRS+A or CRS+CF patients; B. CRS-CF patients are significantly younger than CRS+A patients (ANOVA, Tukey’s p = 0.041); however, no differences in age were observed for pairwise comparisons between the other groups (p > 0.05, Tukey’s post hoc test). Figure S2A. PCoA of an unweighted UniFrac distance matrix colored by DSI-IIIb and healthy (PERMANOVA p = 0.001, 18.2% variation explained); B. PCoA of weighted UniFrac distance matrix after dominant sequence reads associated with the dominant family in each sample were removed demonstrating that DS still significantly explains variation in community composition despite removal of the dominant taxon from each sample (PERMANOVA p = 0.001, 17.6% variation explained). Figure S3A. Laplace model fit demonstrates three distinct Dirichlet multinomial mixtures groups. B. Hierarchical cluster analysis using a weighted-UniFrac distance matrix showing that microbiomes enriched in Corynebacteriaceae forms a distinct cluster (au p = 100). Heatmap shows relative abundance of the bacterial general that comprise >90% of the total sequence reads. C. Reciprocal relationship between Corynebacteriaceae and Staphylococcaceae. Figure S4. PICRUSt-predicted functional variation across microbial Dirichlet states shows significant functional differences A. PCoA of Canberra distance matrix; PERMANOVA p = 0.001, 21.7% of variation explained) B. PCoA of Bray-Curtis distance matrix; PERMANOVA p = 0.001, 22.0% of variation explained). Figure S5. Expression levels of all host immune genes measured by QPCR (*indicates Kruskal-Wallis p < 0.05, q < 0.15; **indicates Kruskal-Wallis p < 0.05, q < 0.05; DS vs. nonCRS). Table S1–S5. (ZIP 1671 kb) [file 40168_2017_266_MOESM6_ESM.zip › FigS4_revised_color040617.pdf]

IL8

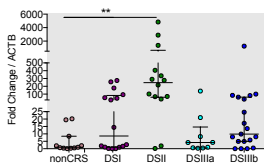

TNF-alpha

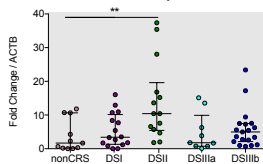

IL1B

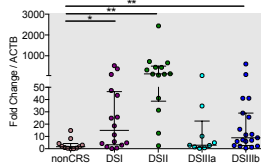

IL6

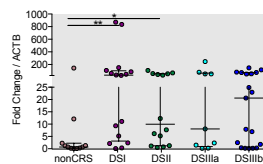

IL10

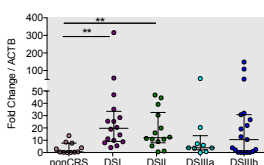

IL5

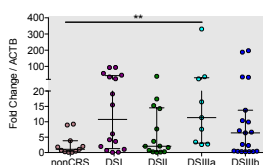

IFN-gamma

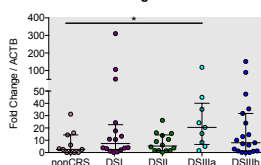

OCEL1

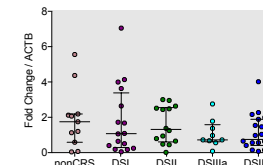

CLDN2

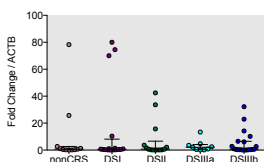

TGFb1

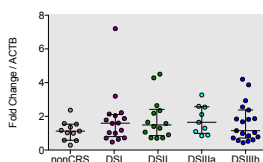

CCL11

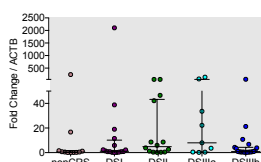

ARG1

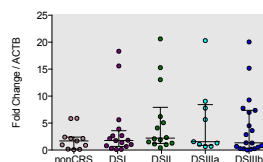

MUC5AC

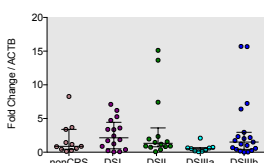

TSLP

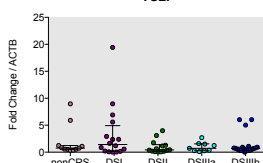

CLCA1

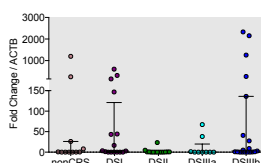

IL25

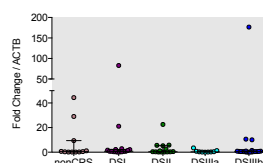

IL4

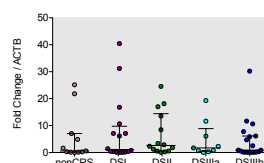

IL13

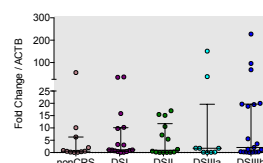

IL17A

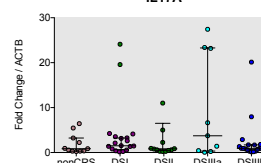

IL33

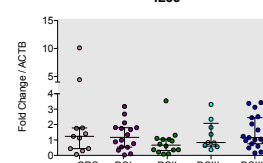

Supplement: Supplementary file 6 — Supplemental Information. Figure S1A. Lund-MacKay scores associated with disease state. No differences were observed between CRS, CRS+A or CRS+CF patients; B. CRS-CF patients are significantly younger than CRS+A patients (ANOVA, Tukey’s p = 0.041); however, no differences in age were observed for pairwise comparisons between the other groups (p > 0.05, Tukey’s post hoc test). Figure S2A. PCoA of an unweighted UniFrac distance matrix colored by DSI-IIIb and healthy (PERMANOVA p = 0.001, 18.2% variation explained); B. PCoA of weighted UniFrac distance matrix after dominant sequence reads associated with the dominant family in each sample were removed demonstrating that DS still significantly explains variation in community composition despite removal of the dominant taxon from each sample (PERMANOVA p = 0.001, 17.6% variation explained). Figure S3A. Laplace model fit demonstrates three distinct Dirichlet multinomial mixtures groups. B. Hierarchical cluster analysis using a weighted-UniFrac distance matrix showing that microbiomes enriched in Corynebacteriaceae forms a distinct cluster (au p = 100). Heatmap shows relative abundance of the bacterial general that comprise >90% of the total sequence reads. C. Reciprocal relationship between Corynebacteriaceae and Staphylococcaceae. Figure S4. PICRUSt-predicted functional variation across microbial Dirichlet states shows significant functional differences A. PCoA of Canberra distance matrix; PERMANOVA p = 0.001, 21.7% of variation explained) B. PCoA of Bray-Curtis distance matrix; PERMANOVA p = 0.001, 22.0% of variation explained). Figure S5. Expression levels of all host immune genes measured by QPCR (*indicates Kruskal-Wallis p < 0.05, q < 0.15; **indicates Kruskal-Wallis p < 0.05, q < 0.05; DS vs. nonCRS). Table S1–S5. (ZIP 1671 kb) [file 40168_2017_266_MOESM6_ESM.zip › FigS5_040617.pdf]
